# Genomic mosaicism due to homoeologous exchange generates extensive phenotypic diversity in nascent allopolyploids

**DOI:** 10.1093/nsr/nwaa277

**Published:** 2020-11-07

**Authors:** Ying Wu, Fan Lin, Yao Zhou, Jie Wang, Shuai Sun, Bin Wang, Zhibin Zhang, Guo Li, Xiuyun Lin, Xutong Wang, Yue Sun, Qianli Dong, Chunming Xu, Lei Gong, Jonathan F Wendel, Zhiwu Zhang, Bao Liu

**Affiliations:** Key Laboratory of Molecular Epigenetics of the Ministry of Education, Northeast Normal University, Changchun 130024, China; Department of Crop & Soil Sciences, Washington State University, Pullman, WA 99164, USA; Brightseed Inc., San Francisco, CA 94107, USA; Department of Crop & Soil Sciences, Washington State University, Pullman, WA 99164, USA; Key Laboratory of Molecular Epigenetics of the Ministry of Education, Northeast Normal University, Changchun 130024, China; Key Laboratory of Molecular Epigenetics of the Ministry of Education, Northeast Normal University, Changchun 130024, China; Key Laboratory of Molecular Epigenetics of the Ministry of Education, Northeast Normal University, Changchun 130024, China; Key Laboratory of Molecular Epigenetics of the Ministry of Education, Northeast Normal University, Changchun 130024, China; Key Laboratory of Molecular Epigenetics of the Ministry of Education, Northeast Normal University, Changchun 130024, China; Key Laboratory of Molecular Epigenetics of the Ministry of Education, Northeast Normal University, Changchun 130024, China; Key Laboratory of Molecular Epigenetics of the Ministry of Education, Northeast Normal University, Changchun 130024, China; Key Laboratory of Molecular Epigenetics of the Ministry of Education, Northeast Normal University, Changchun 130024, China; Key Laboratory of Molecular Epigenetics of the Ministry of Education, Northeast Normal University, Changchun 130024, China; Key Laboratory of Molecular Epigenetics of the Ministry of Education, Northeast Normal University, Changchun 130024, China; Key Laboratory of Molecular Epigenetics of the Ministry of Education, Northeast Normal University, Changchun 130024, China; Department of Ecology, Evolution & Organismal Biology, Iowa State University, Ames, IA 50011, USA; Department of Ecology, Evolution & Organismal Biology, Iowa State University, Ames, IA 50011, USA; Department of Crop & Soil Sciences, Washington State University, Pullman, WA 99164, USA; Key Laboratory of Molecular Epigenetics of the Ministry of Education, Northeast Normal University, Changchun 130024, China

**Keywords:** nascent allopolyploidy, homoeologous recombination, phenotypic diversity, plant evolution, GWAS, dosage effects, epistasis

## Abstract

Allopolyploidy is an important process in plant speciation, yet newly formed allopolyploid species typically suffer from extreme genetic bottlenecks. One escape from this impasse might be homoeologous meiotic pairing, during which homoeologous exchanges (HEs) generate phenotypically variable progeny. However, the immediate genome-wide patterns and resulting phenotypic diversity generated by HEs remain largely unknown. Here, we analyzed the genome composition of 202 phenotyped euploid segmental allopolyploid individuals from the fourth selfed generation following chromosomal doubling of reciprocal F1 hybrids of crosses between rice subspecies, using whole-genome sequencing. We describe rampant occurrence of HEs that, by overcoming incompatibility or conferring superiority of hetero-cytonuclear interactions, generate extensive and individualized genomic mosaicism across the analyzed tetraploids. We show that the resulting homoeolog copy number alteration in tetraploids affects known-function genes and their complex genetic interactions, in the process creating extraordinary phenotypic diversity at the population level following a single initial hybridization. Our results illuminate the immediate genomic landscapes possible in a tetraploid genomic environment, and underscore HE as an important mechanism that fuels rapid phenotypic diversification accompanying the initial stages of allopolyploid evolution.

## INTRODUCTION

Phylogenetic and phylogenomic studies have revealed that hybridization is widespread in all domains of life [1–4]. Merging of genomes from divergent lineages represents a potent evolutionary force that can facilitate adaption, speciation and adaptive radiation [3–6]. There are two major forms of hybridization, one at the homoploid level and the second, allopolyploidization, entailing whole-genome duplication (WGD). Allopolyploidy is pervasive in the evolutionary history of higher plants, testifying to its creative role in adaptive evolution and species diversification of the plant kingdom [7–11]. Compared with newly formed homoploid hybrids that are often, though not always, sterile due to genic and/or chromosomal incompatibility [12], nascent allopolyploids often are partially to fully fertile, because of rapid establishment of diploid-like meiotic behavior [13–15]. It is established that many polyploids occur recurrently from different populations of their progenitors, whereby new genotypes are generated upon secondary contact [16], but some polyploids are of monophyletic origin. Regardless, in nascent allopolyploids, perfect homologous meiotic pairing often generates little variation, thus limiting evolvability [17]. This property of allopolyploidy constrains the generation of genetically variable progeny and also impedes purging of fixed deleterious or slightly deleterious mutations due to genome merger. In addition, *de novo* recessive beneficial mutations that occur post-polyploidy will be masked by genetic redundancy [18]. Nonetheless, the near-ubiquity and prevalence of allopolyploidy across the angiosperm phylogenetic spectrum comprises *prima facie* evidence that there are solutions to the seemingly insurmountable constraints imposed by the foregoing population genetic considerations.

Apart from recurrent formation [16], another mechanism to mitigate allopolyploidy-associated genetic impoverishment is repeated introgression from diploid parental progenitors or related taxa [2,19,20], especially during niche expansion or human-mediated dissemination [15,21–23]. Yet, prior to these extrinsic sources of variation coming into play, how might nascent allopolyploids generate phenotypically relevant variation? At least a partial answer to this question is related to the multiple and diverse mechanisms of rapid changes in the genome, transcriptome and epigenome of allopolyploids [9,24–26]. It should be noted however that these immediate genomic responses due to genome merge and/or doubling turned out to be largely maladaptive in animals, which provides a novel explanation to the long-standing enigma, i.e. why polyploidy is rarer in animals but abundant in plants [27]. Intriguingly, in some lower vertebrates, such as certain fish, these allopolyploidization-incurred catastrophic genome consequences can be resolved by subgenome cooperation and balanced stabilization, and lead to re-diploidized lineages [28].

An important and frequent observation in many plant allopolyploids is that homologous chromosome meiotic pairing is not stringent, and that homoeologous exchanges (HEs) may arise that are transgenerationally cumulative and may be subject to natural and human selections [29–39]. It thus is evident that in many young plant allopolyploids, HEs provide a possible escape from pure homozygosity and that this may be an effective mechanism for generating rapid genetic variation [34]. Relatively little is understood, however, about the dynamics and pace of HE-mediated genomic diversification at the genomic and population levels, and even less is understood about its direct phenotypic consequences in the absence of confounding evolutionary forces.

Here, we focus on a segmental allotetraploid rice (*Oryza sativa*) population consisting of 202 sampled euploid individuals derived from inter-subspecies (*japonica* and *indica*) hybridization and chromosome doubling [40]. Previously, we used this system to assess the association between HEs and partitioning of homoeologous gene expression based on a subset of pre-selected genes [41], and effects of HEs on alternative splicing [42] and on DNA methylation stability [43] at individual plant levels. Here, we extend these analyses to genome-scale and at population levels, with the aims of (i) characterizing the immediate genomic landscape generated by HE-mediated admixture of two divergent genomes following WGD; (ii) determining the features and factors that constrain HE occurrence and/or perpetuation; and (iii) assessing the immediate impact of HE-mediated genomic mosaicism on phenotypic variation, as well as deciphering its underlying genetic basis. We show that (i) rampant HEs occurred in the tetraploids, generating widespread genomic mosaicism; (ii) cytonuclear interaction is an important intrinsic factor that constrains particular admixed patterns; and (iii) the extensive phenotypic diversity in the tetraploids is largely accounted for by HE-mediated homoeolog copy number alteration of known-function large-effect genes and their multiple interactions.

## RESULTS

### Extraordinary phenotypic diversity

**Figure 1.**
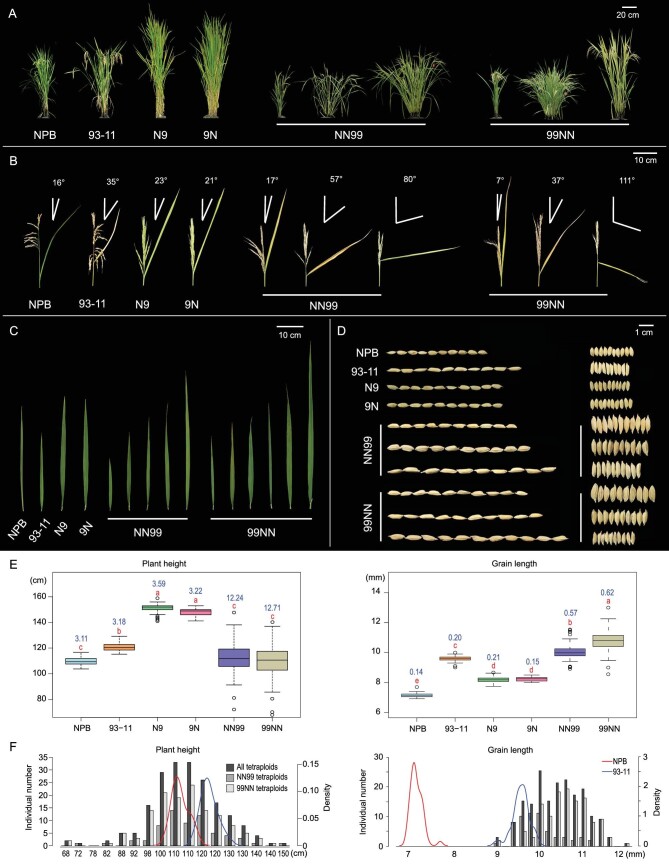
Illustration and quantification of phenotypic traits of the diploid parents (NPB and 93-11), reciprocal F1 hybrids (N9 and 9N) and reciprocal S4 tetraploids (NN99 and 99NN). (A) Overall plant status including plant height, tiller number and tiller angle. (B) Flag-leaf angle. (C) Flag-leaf length and width. (D) Grain length and width. (E) and (F) Quantification of plant height and grain length by boxplots and histograms, respectively. In (D), the aligned seeds depicting variations in grain length (horizontally arranged) and width (vertically arranged) are from different tetraploid lines (the 10 seeds arranged in each row are from one line). In (E), letters above each box denote statistically different phenotypic distributions in each comparison, with blue numbers above each box referring to the relevant standard deviations of the data from each box. In (F), the left ordinates are for the histograms and the right ordinates are for the density plots (the red and blue curves).

We reported previously that the synthetic tetraploids (segmental allotetraploids) [44,45] of the rice subspecies *japonica* (cv. Nipponbare) and *indica* (cv. 93-11) manifested considerable phenotypic novelty compared with their parental cultivars and F1 hybrids. In addition, they displayed extensive changes in gene expression and alternative splicing as a result of the combined effects of hybridization and genome duplication [40–42]. Here we extend these previous results to describe the spectrum of phenotypic variation in progenies of the tetraploids at the population level, and explore their underlying genetic variation based on high-quality whole-genome analyses. We phenotyped 21 complex traits at the fourth selfed generation (S4) tetraploid populations of reciprocal origins (NN99 and 99NN) which contained a set of 202 euploid individuals (Supplementary Fig. S1 and Dataset S1). Populations of both parents and reciprocal F1 hybrids were phenotyped in parallel. Of these 21 traits, nine were related to vegetative growth and development, while the other 12 were related to reproduction and seed yield (Dataset S1–S2). Compared with the parents, F1 hybrids were uniformly heterotic in traits related to vegetative growth but inferior in traits related to seed production (Fig. 1A–D, Supplementary Fig. S2 and Dataset S2); the latter is expected given the inter-subspecific genic incompatibility that causes high hybrid infertility [46].

While the distribution of mean values of tetraploids, irrespective of cross direction, did not exceed either the diploid parents or F1 hybrids in 16 of the 21 traits, it was transgressive relative to the parents and F1 hybrids in the other five traits (Dataset S2). A striking feature of the tetraploids was the magnitude of variation in all 21 traits (Fig. 1A–E, Supplementary Fig. S2–S3 and Dataset S1). Standard deviation (SD), range (R, maximum minus minimum) and coefficient of variation (CV) analyses all confirmed that both tetraploid populations had substantially larger variation than those of the diploid parents and reciprocal F1 hybrids for all 21 traits (Fig. 1E, Supplementary Fig. S3 and Dataset S1). As expected, we found that although allopolyploidization (the combined effects of hybridity and polyploidy) itself contributed to phenotypic differences between the tetraploids and diploid parents and F1 hybrids, this effect cannot cause the rapidly emerged, dramatic variations in each trait among the tetraploids at population levels (Supplementary Fig. S4 and Supplementary Results and Analysis). Because there was no discernible difference in the phenotypic data distribution for reciprocal tetraploid populations (Fig. 1F and Supplementary Fig. S5), we do not differentiate them here unless indicated otherwise.

Notably, transgressive phenotypes were observed for all traits in at least some of the tetraploids (Fig. 1F, Supplementary Fig. S5 and Table S1). The number of individuals manifesting phenotypic over-transgressivity (greater than both parents) was significantly higher than those showing under-transgressivity (smaller than both parents) for 11 of the 21 traits, whereas the reverse was observed for nine traits, and one trait showed no significant difference (Supplementary Table S1).

### Rampant homoeologous exchange

Given the close phylogenetic relatedness between the two rice subspecies, *japonica* and *indica* [47,48], it is expected that meiotic HE may occur in selfed progenies of the tetraploids, as indeed we showed in a pilot study involving four tetraploid individuals [43]. To further quantify the extent of HE at genome-wide scale in progenies of the tetraploids at a population

level, we performed whole-genome re-sequencing (10X coverage) of 202 euploid tetraploids selected from a set of 340 individuals based on oligo-FISH (florescence *in situ* hybridization)-based karyotyping [49] (Supplementary Fig. S6). By using a customized pipeline, we verified the euploid identity of all 202 individuals, and determined the foci of HE breakpoints in each individual at a 5-kilobase (kb) resolution along each of the 12 rice chromosomes. Reliability of our pipeline was also validated by performing the same analysis on whole-genome re-sequencing data of the reciprocal F1 hybrids [43], and in which no recombinant tract (mimicking HE) was detected. Quantification across the 202 re-sequenced individuals (Supplementary Fig. S7) identified a total of 27 945 HEs after only four selfed generations, mapping to all 12 rice chromosomes (mean of 138.34 HEs per individual). This surprisingly large number of HEs may suggest they are not only transgenerationally cumulative but also likely arising in a ‘ratchet-like’ manner [34]. To test this, we analyzed HEs from an additional 45 individuals of the S5 (whole-genome sequencing data available) generation that are direct progenies of random individuals of the 202 S4 tetraploids (Supplementary Fig. S1 and Supplementary Results and Analysis). We found that HE rates per meiosis were 17.4 and 19.0 (or 0.72 and 0.79 per chromosome pair, *n* = 24) in S4 and S5, respectively (*P* = 0.0269, Student's *t*-test), lending support to the ‘polyploid-ratchet-like’ metaphor [34]. However, the ratcheting process may hold only for a limited number of generations, i.e. before the segregating tetraploids reach a certain homozygosity threshold. HE frequencies varied markedly among chromosomes, with 1, 4 and 12 showing larger numbers of HEs while fewer were detected on chromosomes 6, 8 and 10 (Supplementary Table S2). However, when scaled by chromosome size, chromosomes 12 and 6 respectively showed an excess (27.24 cM/Mb, *P* < 0.05) and deficit (10.76 cM/Mb, *P* < 0.05) of HEs relative to expectations based on permutation-based Poisson tests (Supplementary Table S2). The inter-chromosome difference in HE frequency is interesting given that homoeologous recombination is likely under the control of the same machinery as homologous recombination (HR) [50], which primarily acts *in trans* [51]. Nonetheless, similar observations were made in synthetic allotetraploids of *Brassica* [33] and wheat [50], suggesting generality of the phenomenon.

With respect to within-chromosome distribution, a general feature is lower density of HEs in pericentromeric regions (defined as three consecutive 500 kb bins harboring the centromere), while subtelomeric regions (defined as four consecutive 500 kb bins from the end of each chromosomal arm) showed the opposite trend (Supplementary Fig. S7 and Table S3); this observation is consistent with patterns of HR in plants [50], and again, suggests the same recombination machinery is at work [43]. Exceptions to this generality are apparent, however. For example, chromosome 1 experienced more HEs in the pericentromeric region and chromosome 9 showed fewer HEs in the subtelomeric regions (Supplementary Fig. S7 and Table S3). This chromosome-specific peculiarity of HEs was not found for the distribution of homologous recombination in rice [51,53,54], suggesting it is a unique property of HE.

**Figure 2.**
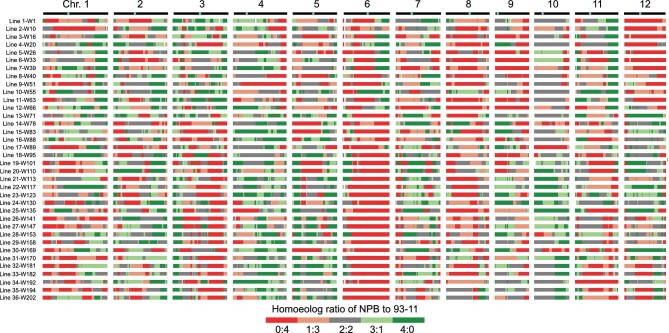
Heatmaps depicting the genomic landscapes of 36 randomly selected S4 euploid tetraploid individuals from 36 selfed lines (Supplementary Fig. S1). Different colors denote different homoeolog compositions, where orange representing NPB homoeolog percentage at a given locus is 25%, i.e. the homoeolog ratio between NPB and 93-11 is 1 : 3. Each row represents one tetraploid individual, the 12 columns represent the 12 chromosomes in the rice genome, and the light blue dots denote centromeres.

For any given locus in an S4 individual, the ratio of homoeologs from the two parents, Nipponbare and 93-11, may fall into one of five types, i.e. Nipponbare:93-11 = 4 : 0, 3 : 1, 2 : 2, 1 : 3 or 0 : 4. We analyzed the genomic composition of all 202 S4 tetraploids and depicted their genomic composition either on a per-individual basis (one random

individual for each of the 36 lines; Fig. 2) or on a per-line basis (all five or six individuals of a given line together; Supplementary Fig. S8). Genome-wide, the proportions of each of the five Nipponbare vs. 93-11 homoeolog ratios of all 202 individuals together were 17.4% (4 : 0), 13.0% (3 : 1), 24.8% (2 : 2), 16.2% (1 : 3) and 28.5% (0 : 4), respectively; notably, proportions between both the homologous ratios (4 : 0 vs. 0 : 4) and the heterozygous ratios (3 : 1 vs. 1 : 3) were asymmetric with respect to the null assumption of 50% : 50% (*P* < 3.4E-16, exact binomial test; Supplementary Table S4). Overall, the genomic proportion of 93-11 homoeologs (averaged 56.34%) was significantly higher than that of Nipponbare (NPB) homoeologs (averaged 43.66%) in the tetraploids (*P* < 0.05, exact binomial test), and this trend holds in both cross directions (*P *= 0.077, chi-square test). Relative proportions of the five homoeolog ratios were also not equal among the 12 chromosomes. When considering together the homozygous (4 : 0 and 0 : 4) and heterozygous (3 : 1, 2 : 2 and 1 : 3) homoeolog ratios each as a group, chromosome 6 showed the highest proportions (average = 80.7%) of homozygous ratios, which were mainly contributed by the 93-11 homoeologs (average = 70.0%) (Supplementary Table S4), while chromosome 10 showed the highest proportion of heterozygous ratios, on average 70.9% (Supplementary Table S4). Chromosomes 6 and 8 were overrepresented by homo-93-11 (NPB: 93-11 = 0 : 4) across the 202 individuals (Fig. 2 and Supplementary Fig. S8). We suspect this biased parental legacy is likely due to selection for early flowering in the northeast region of China where the plants were grown, consistent with enrichment of genes controlling heading date in chromosomes 6 and 8 (https://shigen.nig.ac.jp/rice/oryzabase/and http://www.ricedata.cn/index.htm).

### Homoeologous exchange is constrained by cytonuclear interaction

**Figure 3.**
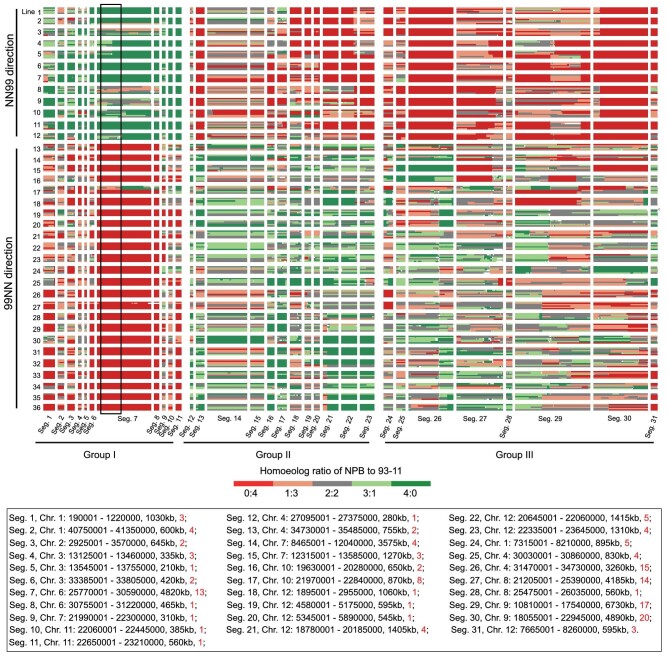
Heatmaps showing the genomic compositions of 31 cytonuclear compatibility-related chromosomal segments being strongly selected for from all of the 202 genome-re-sequenced S_4_ tetraploid individuals. The different colors show the different genomic types, e.g. the orange color represents that the homoeologous ratio between NPB and 93-11 in this given locus is 1 : 3. Each row represents one tetraploid individual, the 31 columns indicate 31 chromosomal segments. Cross direction and line names are labeled on the left of the rows. Information of segment ID, chromosome, genomic location, segment size and the number of cytonuclear molecular interaction genes is given in the lower panel. The framed box in Seg. 7 denotes a 1787 kb genomic region retaining at least one maternal copy in all (100%) individuals.

A salient observation is that some genomic regions manifested parental homoeolog composition patterns that are strikingly distinct between the tetraploid reciprocals, suggesting the possibility of hetero-cytonuclear incompatibility or superiority (Fig. 3). Genomic regions showing such features could be classified into three groups: Group I contained 11 segments (segments 1 to 11) that mapped to six chromosomes (1, 2, 3, 6, 7 and 11), with sizes ranging from 210 to 1030 kb, in which at least one copy of the maternal homolog was preferentially retained (*P* < 0.01, chi-square test), in a reciprocal manner, in >95% individuals, suggesting symmetric hetero-cytonuclear incompatibility. Group II contained 12 segments (segments 12 to 23) that mapped to four chromosomes (4, 7, 10 and 12) with sizes ranging from 280 to 1310 kb, in which at least one copy of paternal homolog was preferentially retained (*P* < 0.01, chi-square test), in a reciprocal manner, in >95% individuals, suggesting symmetric hetero-cytonuclear superiority. Group III contained eight segments (segments 24 to 31) that mapped to five chromosomes (1, 4, 8, 9 and 12) with sizes ranging from 560 to 6730 kb, which showed preferential retention (*P* < 0.01, chi-square test) of at least one copy of the paternal homoeolog in all individuals of the NN99 but not in 99NN (Fig. 3), suggesting asymmetric hetero-cytonuclear superiority. Notably, although hetero-cytonuclear incompatibility (group I) did not involve 100% of the individuals, the 5% of plants that did not harbor homo-cytonuclear compositions showed significant loss in reproductive fitness (i.e. fecundity) compared to their respective siblings of the 95%, reflected by reduced fertility (36.4% vs. 76.0%, *P* = 1.13E-05, Student's *t*-test) and grain number per panicle (52.3 vs.107.3, *P *= 0.0096, Student's *t*-test) (Supplementary Table S5).

There were 1529, 2029 and 3517 genes that mapped to the segments of Groups I, II and III, respectively (Dataset S3A–B). Although gene ontology (GO) enrichment analysis of all three sets of genes, either together or separately, showed no specific functional relevance (Dataset S3C), we noted that each of the 31 segments harbored at least one gene that was functionally categorized as participating in cytonuclear (plastid- or mitochondrion-nuclear) molecular interactions, and in total, 147 such genes were identified (Fig. 3 and Dataset S3A and D). Specifically, groups I, II and III contained 32, 36 and 79 such cytonuclear interacting genes, respectively, which are significantly more than expected from the genome-wide average (Pearson's Chi-squared test: *P* = 1.02E-4, 5.74E-3 and 1.32E-11, for groups I, II and III, separately, and *P* = 3.32E-14 for all three groups in aggregate) (Supplementary Table S6). Of these 147 genes, 21 participate in cytonuclear co-encoding enzyme complexes (CCECs), in which different subunits of organellar protein complexes are encoded by organellar (mitochondrion or plastid) and nuclear genes, while 126 encode cytonuclear enzyme complexes (CECs; organelle-targeting proteins without organellar interacting partners) (Dataset S3A and D). Notably, 103 of the 147 genes (collectively on all 31 segments) showed predicted functional divergence between the parental alleles (Dataset S3D). This suggests that biased retention might be functionally consequential. Taken together, these results suggest that both hetero-cytonuclear incompatibility and superiority likely are constraints that have contributed to homoeologous composition of the tetraploids, either symmetrically (both cross directions are affected) or asymmetrically (only one direction is affected).

### Homoeologous expression is predominantly copy number-dependent

One immediate genetic consequence of HE is disruption of homoeologous expression ratios determined by parental legacy. Conceivably, for homoeologs that are functionally diverged or sub-functionalized between the parents, this outcome of HEs may have physiological and phenotypic consequences if expression levels correlate with copy number [55]. To address this, we performed transcriptome profiling using two tissues (leaf and root) sampled from 12 randomly selected S4 tetraploid individuals. From 11 761 to 13 800 and from 13 892 to 15 511 expressed genes were identified in leaf and root, respectively, across the 12 tetraploid individuals (Supplementary Table S7). Most genes (ca. 90%) showed a strong correlation between ratios of homoeolog transcript abundance and ratios of DNA homoeolog copy number, in both leaf and root (adjusted *P* < 0.05 by chi-square test; Supplementary Fig. S9 and Table S7). This indicates homoeologous expression levels for most genes in the rice tetraploids are dosage-sensitive and homoeolog copy number-dependent, likely due to constraint for total expression level to maintain gene balance [56,57]. This is also consistent with the recently documented regulatory evolutionary features of the rice genome, and their important fitness consequences [58].

### HEs include characterized large-effect genes that underpin trait variation

To pinpoint the specific HE-mediated copy number variants that may be responsible for phenotypic diversity, we performed a genome-wide association study (GWAS) between variation in genome-wide homoeolog ratio and variation in each of the 21 quantified traits. The fixed and random model circulating probability unification (FarmCPU) method of GWAS was used to maximize statistical power and robustness [59]. We used both additive and dominance models of GWAS and encoded the five types of homoeolog ratios (N : 9 of 0 : 4, 1 : 3, 2 : 2, 3 : 1 and 4 : 0) as 0, 1, 2, 3 and 4, respectively for the additive model. For the dominance model, we used three types of coding for the five homoeolog ratios corresponding to N : 9 of 0 : 4, 1 : 3, 2 : 2, 3 : 1 and 4 : 0, namely, (i) 0, 1, 1, 1, 0; (ii) 0, 2, 2, 2, 1; and (iii) 1, 2, 2, 2, 0. This was to reflect differential effects of the parental homoeologs when they had alternative homozygous (4 : 0 and 0 : 4) states, i.e. (i), (ii) and (iii) reflect equivalence, transgressive N and transgressive 9, respectively, while the three heterozygous homoeolog ratios (1 : 3, 2 : 2, 3 : 1) were coded as 1 or 2 to reflect transgressive phenotypic values in both directions. To eliminate potential false positives, we used the most conservative threshold, Bonferroni corrections; the threshold for significant association calling was determined to be *P* < 1.3395E-07.

There were 22 and 63 distinct signals passing the statistical threshold in 14 and 21 traits using the additive and dominance models, respectively (Supporting Material and Dataset S4A–V). Notably, some signals were detected in both models, suggesting they have both additive and dominant effects on the target traits. For each GWAS-reported signal, sizes of linked segments were decided by Pearson correlation analysis (correlation coefficient *r* > 0.9; Dataset S4B–V). Next, we scrutinized known genes located within the identified segments. In total, we identified 29 known genes in these segments, which were previously shown as causally linked to the traits (Dataset S4A–V). These include large-effect genes such as *GS3* for grain length [60], *TAC1* for tiller angle [61,62], *NAL1* for flag-leaf width [63,64] and *DTH7* for days to flowering [65]. Notably, these four genes are also responsible for trait divergence between Nipponbare and 93-11 (Supplementary Table S8, Supplementary Results and Analysis and Dataset S4B, E, G and S). Although no information regarding functional divergence between the two rice subspecies is available for the other 25 genes, we found 21 bear non-synonymous coding differences between the parents, suggestive of protein-level functional diversification (Dataset S4–V).

**Figure 4.**
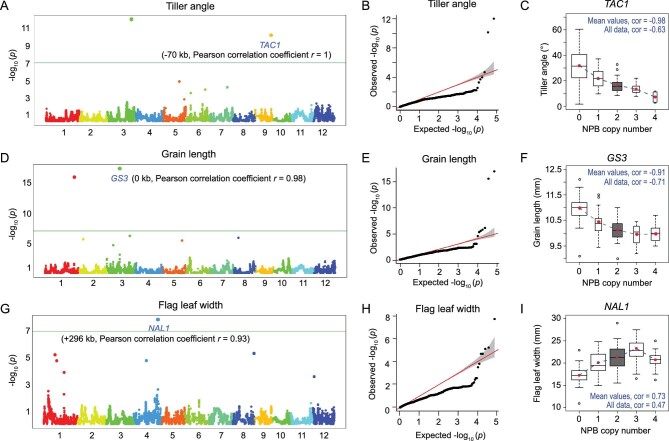
GWAS of tiller angle, grain length and flag-leaf width with the additive model by FarmCPU R scripts. (A), (D) and (G) are Manhattan plots, wherein the green lines represent thresholds based on Bonferroni tests, with genes controlling the given trait labeled in blue below the corresponding locus. (B), (E) and (H) are Quantile-Quantile plots of p-values. (C), (F) and (I) are boxplots showing the additive relationship between phenotype and NPB copy number in each of the three genes.

As an illustration, we show the associations between homoeolog ratios of three genes (*GS3*, *TAC1* and *NAL1*) with their corresponding traits (grain length, tiller angle and flag-leaf width) revealed by the additive model in GWAS, using both Manhattan (Fig. 4A, D and G), Quantile-Quantile (QQ) plots (Fig. 4B, E and H) and box blots (Fig. 4C, F and I). For the *TAC1*-containing locus, if the balanced heterozygous state N : 9 = 2 : 2 is the starting point, tiller angle becomes larger as NPB homoeolog copy number decreases (hence proportional increase of the 93-11 homoeolog copy number) and *vice versa* (Fig. 4C). This mirroring phenotypic response indicates a negative linear correlation (*r* = −0.63, *P* < 2.2E-16, Pearson correlation test) between NPB homoeolog copy number of *TAC1* and tiller angle in the tetraploids, and hence additive effects of the gene. A similar negative linear correlation (*r* = −0.71, *P* < 2.2E-16 by Pearson correlation test) was revealed between NPB homoeolog copy number of the *GS3*-containing locus and grain length, although in this case there was a slight deviation from the expected result when the NPB homoeolog copy number was 4, suggesting, in addition to its major additive effect, that there also existed a moderate dominant and/or epistatic effect (Fig. 4F) consistent with the result that *GS3* was identified by both additive and dominant models. For the *NAL1*-containing locus, in principle the NPB homoeolog copy number should be negatively correlated with flag-leaf width since 93-11 has a wider flag-leaf than NPB; unexpectedly, however, we found the correlation was positive in the tetraploids when the copy number of the NPB homoeolog of this locus was in the range of 0 to 3 but not when it reached 4 (*r* = 0.47, *P* = 3.79E-1, Pearson correlation test; Fig. 4I). This again indicates that *NAL1* has additive, dominant and/or epistatic effects, consistent with its detection under both models.

Under the dominance model, we selected three loci (*DTH8*, *OsBZR1* or *OsSIZ1*) as examples to show the associations between homoeolog ratios and the three corresponding traits (days to flowering, thousand kernel weight and tiller number, respectively). These associations are illustrated using Manhattan plots (Supplementary Fig. S10A, D and G), QQ plots (Supplementary Fig. S10B, E and H) and boxplots (Supplementary Fig. S10C, F and I). For the *DTH8*-containing locus identified in the 0-1-1-1-0 dominance model, tetraploids with heterozygous homoeolog ratios showed significantly fewer days to flowering than those with homozygous homoeolog ratios; however, no difference in the trait was evident among the three heterozygous homoeolog ratios, suggesting dosage-independent interaction (Supplementary Fig. S10C). This strong curvilinear association (*r* = −0.29, *P* = 6.8E-05, Pearson correlation test) points to a negative dominant effect of the *DTH8*-containing locus on days to flowering. A similar negative dominant association (*r* = −0.29, *P* = 4.8E-05, Pearson correlation test) between the *OsBZR1*-containing locus fitting the 1-2-2-2-0 model of NPB homoeolog copy number and thousand kernel weight was identified, although in this case it appeared the 2 : 2 heterozygous ratio had a stronger effect than the 3 : 1 or 1 : 3 heterozygous ratio, suggesting dosage-dependent interaction (Supplementary Fig. S10F). In contrast, a positive dominant association (*r* = 0.34, *P* = 1.4E-06, Pearson correlation test) between the *OsSIZ1-*containing locus and tiller number was detected, i.e. plants bearing all three types of heterozygous homoeolog ratios showed significantly more tiller numbers than those with either homozygous homoeologs, which also represents dosage-independent interaction (Supplementary Fig. S10I). Pairwise sequence comparison indicated that all three known genes, *DH8*, *OsBZR1* and *OsSIZ1*, contain non-synonymous coding differences between the Nipponbare and 93-11 parental alleles (Dataset S4B), suggesting their potential functional divergence.

### Epistasis between different HEs is common

Epistasis, i.e. non-additive interactions between non-allelic genes is widespread in diverse

organisms [66–68] (Supplementary Results and Analysis). Whether different parental homoeolog ratio alterations in a polyploid epistatically interact for a given phenotypic trait has not been reported before. Here we used trait-associated HEs by GWAS in the rice tetraploids and identified 2489 interacting locus pairs for the 21 traits, of which 816 (32.8%) showed significant epistatic effects (Dataset S5A,C–W). The epistatic effects fell into one or more of the four models: additive by additive (A by A), additive by dominant (A by D), dominant by additive (D by A) and dominant by dominant (D by D) (Supplementary Results and Analysis). Frequencies of trait-associated pairwise loci manifesting epistasis were unequal among the traits. For example, flag-leaf length showed the highest percentage of epistatic locus pairs (53.6%), while yield showed the lowest (4.4%) (Supplementary Table S9). Also, not all of the four models of epistasis occurred equally; frequencies of A by A, D by A, A by D and D by D were 10.4%, 9.6%, 7.2% and 5.6% (*P* < 0.05, chi-square test; Supplementary Table S9). A further pathway (KEGG) analysis for genes located in the HE-affected fragments that showed epistasis also implicates enriched pathways known to be involved in the target traits (Supplementary Results and Analysis and Dataset S5B).

**Figure 5.**
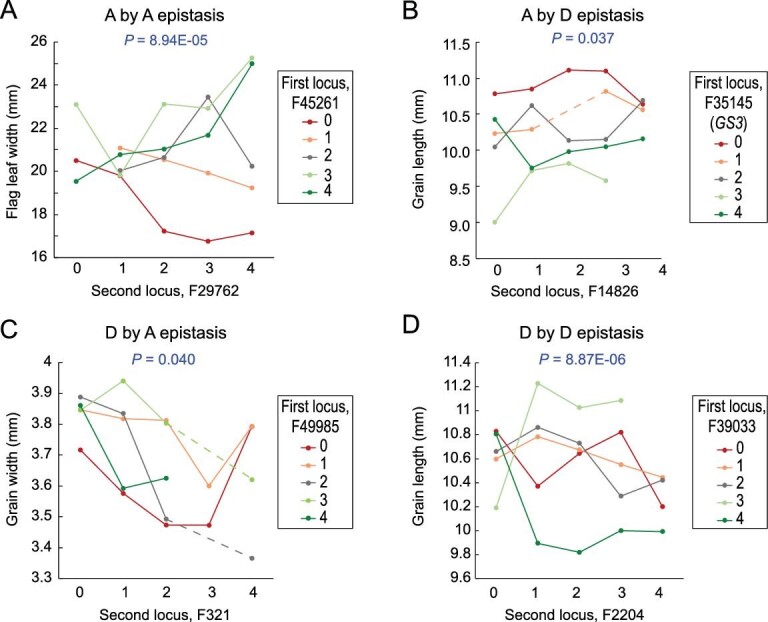
Patterns of interactions of the four epistasis types displayed by various two-locus combinations. *P * value in each panel represents the statistical probability for relative epistasis. The vertical axis represents phenotypic value, and genomic types of 0–4 represent N : 9 homoeolog ratios of 0 : 4, 1 : 3, 2 : 2, 3 : 1 and 4 : 0, respectively.

Each of the four models of epistasis manifested by a pair of loci associated with a typical trait is illustrated in Fig. 5. Individually, both locus F45261 (chromosome 4) and locus F29762 (chromosome 2) showed additive effects on flag-leaf width (Dataset S5F). However, as a pair, these genes showed an A by A epistatic interaction (Fig. 5A). Specifically, F45261 manifested opposite effects when the Nipponbare homoeolog of F29762 had zero and four copies, respectively, and an interdependent effect was evident that scales with Nipponbare homoeolog copy number (Fig. 5A). The locus pairs F35145 (containing *GS3*, chromosome 3) and F14826 (chromosome 11), and F49985 (chromosome 5) and F321 (chromosome 1) exhibited A by D and D by A epistatic interactions on grain length and grain width, respectively (Fig. 5B and C). Specifically, the dominant effects of the Nipponbare homoeolog copy number of F14826 on grain length were incrementally influenced by that of F35145 (Fig. 5B), while the additive effects of the Nipponbare homoeolog copy number of F321 on grain width were dependent on a heterozygous homoeolog state of F49985 (Fig. 5C). The locus pair F39033 (chromosome 3) and F2204 (chromosome 1) showed a D by D epistatic interaction, because the effect of the Nipponbare homoeolog copy number of F2204 on grain length was contingent on F39033 being in a heterozygous homoeolog state, and the two loci interacted more favorably when both homoeologs were heterozygous (Fig. 5D).

## DISCUSSION

Allopolyploidy is widely recognized as a driving force in evolution, most notoriously in plants but also in many other eukaryotic lineages [8,9,69–72]. In many allopolyploids, homoeologous pairing, a prerequisite for HE, is suppressed due to intrinsic parental genome divergence and/or by genetic controls, e.g. *Ph1* in polyploid wheat [73] and *PrBn* in *Brassica* [74], resulting in near exclusive homologous chromosome pairing. This diploid-like meiotic behavior exhibited by many allopolyploids confers genome stability and organismal fertility, yet it may constrain evolvability due to the chromosomal homogeneity of offspring, especially during the initial stages of nascent allopolyploidy. HE was first systematically studied in *Brassica* synthetic allotetraploids using DNA markers [33,75], and proposed as the root cause directly or indirectly underlying rapid genomic and gene expression changes, as well as phenotypic novelty widely reported in nascent plant allopolyploids [34]. Many established allopolyploid species have genomes that have undergone HEs, as evidenced by a large number of recently studied allopolyploid crops and wild species [29,31,35–38,76–79]. In all cases in which the consequences of HEs have been studied, they have been found to alter gene expression and/or phenotypes, suggesting that HE is a powerful force for generating diversity in allopolyploidy [34]. Notably, however, all prior studies at genome-scale are on established species, thus the direct effects of HE cannot be de-convoluted from confounding effects of additional evolutionary forces. Here, by genome re-sequencing of 202 newly synthesized rice tetraploids of pure parental lines of subspecies, which were only four generations old, we unequivocally show that the process of HE can rapidly generate an enormous amount of genomic variation due to HE among derived lines, each of which is unique and carries homoeologs from the two parents that either have become fixed (0 : 4 or 4 : 0) or which will continue to segregate in progenies until they ultimately reach fixation. Thus, HE is a mutagenic mechanism with dual properties, one that generates a massive amount of potentially relevant phenotypic variation following the reunion of two diverged genomes in a common nucleus, but which also will subside (via selfing) as derivative lineages become homozygous for alternative and highly variable suites of homoeologs.

We show that the HEs are genomically widespread but heterogeneous within and among chromosomes, largely but not wholly in line with previous work on the distribution of homologous meiotic recombination [51,52]. Using copy number-dependent homoeolog expression as a foundation, a GWAS identified outlier loci that harbor large-effect known-function genes that are causally associated with the phenotypic traits. We further uncovered that these genes exerted their phenotypic impacts via all possible effects, additive, dominant and epistatic, suggesting their functional connectivity in determining quantitative traits [55]. Most importantly, we demonstrate that the genomic diversity among the S4 lineages has numerous phenotypic consequences, some of which involve traits that could be highly visible to natural (such as flowering time) and human (such as seed size) selections [33,75]. An added dimension to this discovery is that segregating tetraploid plants often exhibited phenotypes that are transgressive relative to the two parents, further increasing the net phenotypic space that might be ‘genomically explored’ during the early stages of an allopolyploid radiation. One can readily envision how selection might shape this diversity in response to varying ecological conditions or changing environments, leading to HE-mediated phenotypic and ultimately taxonomic diversification in allopolyploid lineages as they spread in time and space. HEs thus comprise one powerful means by which allopolyploidy is a creative force for generating biodiversity [34].

One novel aspect of our results concerns the demonstration that cytonuclear co-evolutionary divergence among progenitor diploids may have evolutionary consequences in their derivative allopolyploids, thus adding to our growing appreciation of the cytonuclear dimension of allopolyploid evolution [80]. We show that HE-mediated changes in homo-cytonuclear combinations are preferentially retained reciprocal in the tetraploids, as expected. Unexpectedly, however, large numbers of hetero-cytonuclear combinations were also favorably retained either reciprocally or unidirectionally. While the full scope of the phenotypic consequences of this novel form of cytonuclear selection remain to be studied, it seems clear that this form of interaction may also be important to the evolution of young allopolyploid lineages. Exploring the functional nature of these HE-mediated cytonuclear combinations will likely be a fruitful avenue of future exploration.

In sum, our study documents rapid transgenerational precipitation of extraordinary population genomic heterogeneity subsequent to genome admixture mediated by HE in synthetic plant tetraploids. The extensive yet individualized genomic mosaicism generates wide-ranging population-level phenotypic diversity. Remarkably, much of the phenotypic variation can be readily explained by HE-mediated homoeolog copy number alteration and interaction of large-effect known-function genes. We reveal that cytonuclear interaction, including both homo- and hetero-combinations, is an important constraint underpinning genomic composition of the tetraploids. Our genome-scale and sequence level results demonstrate how HE can be a potent mechanism to rapidly augment the genotypic and phenotypic space of newly formed allopolyploids even parented by pure lines, which provides novel insights into evolvability of nascent allopolyploidy, and bears implications for the rapid generation of genetic and biological diversity of potential contemporary utility.

## CONCLUSION

Classical genetic theory predicts that a solo allopolyploidization event may lead to genetic depauperation due to founder-effect and diploid-like meiotic behavior, and hence is likely maladaptive. This tenet has been refuted by the vast genomic data and our enhanced understanding of polyploid genome evolution. The present study shows that when hybridizing parents are of moderate genetic divergence, allopolyploidization represents a highly permissive arena to enable HE as a major player that catalyzes rampant reshuffling of parental genomes whereby both genotypic and phenotypic space can be massively enlarged. This study provides novel insights with respect to how evolvability of nascent allopolyploidy can be boosted by HE, which also bears implications for translational evolutionary biology for rapid generation of potentially useful biodiversity.

## METHODS

### Plant materials and phenotyping

The rice allotetraploids (NN99 and 99NN) were generated by colchicine treatment of tillers of the reciprocal F1 hybrids (N9 and 9N) between pure line cultivars Nipponbare and 93-11, representing *japonica* and *indica* subspecies of *O.sativa* L. [40]. We term these segmental allopolyploids because of their patterns of chromosomal pairing and divergence, using multiple criteria [39,45,81]. The reciprocal tetraploids used in this study were from colchicine-doubled S0 tetraploids from one tiller of one F1 hybrid individual of each crossing direction, and then selfed for four successive generations, which contained 202 euploid individuals (Supplementary Fig. S1 and Dataset S1). In total, 21 quantitative traits were phenotyped on plants grown in season under paddy-field conditions following standard methods [82]. Details of the morphological data comparisons and statistics are described in Supplementary Materials and Methods.

### Dual-color Oligo-FISH

Two sets of rice oligo libraries were labeled with FAM-green and Texas red using a direct labeling protocol [49]. FISH was performed as reported [83]. Slides were examined under an Olympus fluorescence microscope and digitally photographed.

### DNA and RNA extraction, sequencing and data analyses

Leaves were used for DNA extraction and whole-genome re-sequencing, and both leaves and roots were used for RNA extraction and RNA-seq. Library construction and sequencing were performed by standard Illumina protocols. Detailed information of nucleic acid extraction, sequencing procedure and preliminary data analyses are described in Supplementary Materials and Methods.

### Bioinformatic analysis

Single nucleotide polymorphism (SNP)-based methods were used to determine the genomic compositions and HE loci in all S4 (*n* = 202) and S5 (*n *= 45) tetraploids**.** HE differences among and within chromosomes were tested by using corresponding statistical approaches. Homoeologous transcript ratios between Nipponbare and 93-11 for each gene in two tissues were quantified for homoeologous expression analysis. TargetP [84] (version 2.0) and LOCALIZER [85] (version 1.0.4) were used for genome-wide identification of cytonuclear molecular interaction genes. The online PANTHER 15.0 platform (http://www.pantherdb.org/) was used for GO analysis. KEGG analysis was conducted by the Clusterprofiler package [86] in R program (version 3.4.3, 13). Detailed analysis procedures are described in Supplementary Materials and Methods.

### Genome-wide association study

Existing GWAS pipelines are primarily designed for diploid populations with genome-wide SNPs as the genetic variable to identify the causal locus or loci for a given phenotypic trait. By contrast, in the GWAS of our tetraploid population, the genetic variable is the HE-mediated homoeolog copy number variation (HCNV) of chromosomal segments that harbor large-effect genes with known functions. As a segregating, self-propagating population (S4), there exist five states of HCNV (N : 9 = 0 : 4, 1 : 3, 2 : 2, 3 : 1 and 4 : 0 for a given locus) in the rice allotetraploid populations. Accordingly, the FarmCPU method of GWAS was used [59]. Detailed analysis procedures for GWAS are described in Supplementary Materials and Methods.

### Analysis of epistasis

Pairwise interaction between loci containing trait-determining genes were analyzed by F∞ model [87]. Detailed analysis procedures for epistasis analysis are described in Supplementary Materials and Methods.

### Statistics

All statistical tests in this study were performed using basic packages in R (Version 3.6.1, https://www.r-project.org).

## DATA AVAILABILITY

Clean data for all genome re-sequencing and RNA-seq generated in this study have been deposited in the Sequence Read Archive (https://www.ncbi.nlm.nih.gov/sra) with the accession code PRJNA678613, and the scripts used for data analysis are available at https://github.com/wuying003/HE-identification-for-allotetraploid-rice.

## Supplementary Material

nwaa277_Supplemental_File
