# Supplementary material for: Genomic mosaicism due to homoeologous exchange generates extensive phenotypic diversity in nascent allopolyploids
Source: Natl Sci Rev. 2020 Nov 7;8(5):nwaa277. doi: 10.1093/nsr/nwaa277 (PMC8288387; doi:10.1093/nsr/nwaa277)
Supplement: nwaa277_Supplemental_File [file nwaa277_supplemental_file.zip › Wu et al. Supplementary Data for NSR_MS-2020-1074 Revised.docx]

**Supplementary Data for**

**Genomic Mosaicism due to Homoeologous Exchange Generates Extensive Phenotypic Diversity in Nascent Allopolyploids**

Ying Wu^1,2^, Fan Lin^3^, Yao Zhou^2^, Jie Wang^1^, Shuai Sun^1^, Bin Wang^1^, Zhibin Zhang^1^, Guo Li^1^, Xiuyun Lin^1^, Xutong Wang^1^, Yue Sun^1^, Qianli Dong^1^, Chunming Xu^1^, Lei Gong^1,4,^*, Jonathan F. Wendel^4,^*, Zhiwu Zhang^2,^* and Bao Liu^1,^*

^1^Key Laboratory of Molecular Epigenetics of the Ministry of Education (MOE), Northeast Normal University, Changchun 130024, China; ^2^Department of Crop & Soil Sciences, Washington State University, Pullman, WA 99164, USA; ^3^Brightseed Inc., San Francisco, CA 94107, USA; ^4^Department of Ecology, Evolution & Organismal Biology, Iowa State University, Ames, IA 50011, USA

**∗Corresponding authors.** E-mails: [gongl100@nenu.edu.cn](mailto:gongl100@nenu.edu.cn); jfw@iastate.edu; [zhiwu.zhang@wsu.edu](mailto:zhiwu.zhang@wsu.edu); [baoliu@nenu.edu.cn](mailto:baoliu@nenu.edu.cn)

**This PDF file includes:**

Supplementary Materials and Methods

Supplementary Results and Analysis

Supplementary References

Figures S1 to S10

Tables S1 to S9

Supporting Material

Legends for Datasets S1 to S6

**Other supplementary materials for this manuscript include the following:**

Datasets S1 to S6

**Supplementary Materials and Methods**

**Phenotyping of seed-related traits in three additional control populations.** To further demonstrate that homoeologous exchanges (HEs) are causal to the remarkable phenotypic variability in the S4 tetraploid populations, we phenotyped two seed-related traits, grain length and grain width, of three additional control populations. These populations are: autotetraploids of the two parents, Nipponbare and 93-11 (denoted as NPB-4X and 93-11-4X), the immediate generation (S0) allotetraploids (doubled F1 hybrids) and the 1^st^ selfed generation (S1) allotetraploids.

**Morphological data statistics.** Population level comparisons of phenotypes between genotypes were performed using ANOVA followed by Tukey HSD tests. For parental populations, the upper threshold (95% probability) of the higher-performing parent and lower threshold (5% probability) of the lower-performing parent in each trait were used as the cutoffs to define over- and under-transgressivity of tetraploid individuals, respectively.

**DNA and RNA extraction, sequencing and data analyses.** Genomic DNA was isolated from leaves of the 202 S4 and 45 S5 tetraploid individuals using a modified CTAB method [1] followed by repeated phenol extractions. DNA libraries were subjected to whole genome re-sequencing using the Illumina HiSeq 2500 (125 bp paired end) and NovaSeq 6000 (150 bp paired end) platforms for S4 and S5 individuals, respectively. At least four gigabase (Gb) of clean reads were obtained for each sample. Expanded leaves and roots of 12 randomly chosen S4 tetraploids were sampled to extract total RNA by Trizol according to the manufacturer’s protocol (Invitrogen). RNA libraries were sequenced using the Illumina Xten platform. Four Gb of clean reads were obtained for each sample. For both re-sequencing and RNA-seq data, BWA [2] with -n 5 and STAR [3] with default parameters were used for read-mapping against a modified MSU7.0 Nipponbare genome sequence [4], respectively, in which all sequence differences between NPB and 93-11 were changed to N. GATK [5], was used for SNP calling.

**Determination of genomic composition and HE loci in tetraploids.** The recently reported HE determining protocol [6] was followed with minor modifications. These include (i) homoeolog ratios between Nipponbare and 93-11 were calculated using 5 kb windows; (ii) consecutive windows (n ≥ 10, i.e., 50 kb) showing the same ratio were merged and retained, and sporadic windows showing unexpected ratios were removed; (iii) segments ≤ 200 kb that located in-between two genomic segments with the same homoeolog ratio were removed to avoid biased determination of HEs. Only the previously confirmed reliable SNPs between NPB and 93-11 genomes [7] were used to determine the genomic composition and HE loci in these 202 euploid tetraploids. In addition, to test the reliability of our HE identification pipeline, we further used the resequencing data of the same reciprocal F1 hybrids from our previous study [8] (SRA data accession numbers were SRR8427222 and SRR8427221 in NCBI for N9 and 9N, respectively) with the same depth as the tetraploids (4Gb data was intercepted randomly for each direction F1 hybrid) as negative controls; we did not detect any recombinant tracts (mimicking HEs) in the F1 hybrids of both directions by using the same HE identification pipeline.

**Analyses of HE frequencies among and within chromosomes.** Possible differences in average HE frequencies among the 12 rice chromosomes were tested by upper and lower thresholds (95% and 5% probability, respectively) against 10,000 permutations based on the Poisson distribution. For each chromosome, the pericentromeric and subtelomeric regions were defined as three consecutive 500 kb windows harboring the centromere and four consecutive 500 kb windows from the end of each arm, respectively. A series of customized Perl and R scripts were used to quantify the number of HEs in every 500 kb window along each chromosome and regional differences were analyzed by Student's t-test.

**Homoeologous expression analysis.** Homoeologous transcript ratios between Nipponbare and 93-11 for each gene were quantified by a customized Perl script with the rice genome annotation information based on the vcf file generated from each of the 24 RNA samples (12 of leaf and 12 of root) , and further interrogated by the samtools mpileup program [9]. DNA homoeolog ratios between NPB and 93-11 for each gene of the same plants were generated in parallel. Chi-square tests were used to determine if a given gene shows DNA copy number independent expression with a threshold adjusted *P*-value < 0.05

**Genome-wide identification of cytonuclear molecular interaction genes.** The list of cytonuclear molecular interaction genes in *Arabidopsis thaliana* was downloaded from CyMIRA [10] (<http://cymira.colostate.edu/>). The rice ortholog(s) for each of these *A. thaliana* genes were inferred based on BlastP analysis. Both targetP [11] (version 2.0) and LOCALIZER [12] (version 1.0.4) were used to determine the presence of transit peptides (N-terminal targeting peptides, mTPs and CTPs) in the identified rice genes, and only those with transit peptides reported by both target and LOCALIZER were considered as rice cytonuclear molecular interaction genes.

**Predicting functional divergence between parental (Nipponbare and 93-11) alleles.** Parental differences at the amino acid level for each ortholog pair were analyzed by both all vs. all BlastP and Reciprocal Best Hits (RBH). For motif analysis, HMMER was used for functional domain annotation from Pfam database v31.0 (<http://pfam.xfam.org/>) with an e-value threshold of 1E-05.

**Gene ontology (GO) analysis.** GO analysis was performed using the online PANTHER 15.0 platform (<http://www.pantherdb.org/>) and the molecular function category. GO terms with FDR-corrected *P*-value < 0.05 were considered as overrepresented by Fisher tests.

**Genome-wide association study (GWAS).** The fixed and random model circulating probability unification (FarmCPU) method of GWAS was used [13]. Before performing GWAS, population structure of the tetraploid population was analyzed by Principal Component Analysis (PCA) using the re-sequencing data of all 202 tetraploids. Since reciprocal directions (NN99 and 99NN) of the 202 tetraploids fell into two major groups in the first three PCs (Supplementary Fig. S11A), which explained > 60% of the genomic variance (Supplementary Fig. S11B), group information and the first three PCs were taken as covariates into the GWAS model. For the additive model, five homoeolog ratios of Nipponbare vs. 93-11, i.e., 0:4, 1:3, 2:2, 3:1 and 4:0, were encoded as 0, 1, 2, 3 and 4, respectively. Three dominant models were used, in which the homoeolog ratios 0, 1, 2, 3, 4 were encoded as 0, 1, 1, 1, 0 or 0, 2, 2, 2, 1 or 1, 2, 2, 2, 0 by customized R scripts. The threshold for significant association calling was determined to be *P* < 1.3395E-07 by Bonferroni corrections.

**Analysis of epistasis.** Pairwise interaction between loci containing trait-determining genes were analyzed by F∞ model [14]. A tetraploid F∞ model (Dataset S6) was formulated based on this. For each locus, the homoeolog ratio of N:9 has 5 genotypes: 0, 1, 2, 3 and 4. A total of 25 genotypic combinations were included in the model. We fit a baseline model with the effects of individual genes and then added four types of interaction effect separately: A by A (Additive X Additive), A by D (Additive X Dominant), D by A (Dominant X Additive) and D by D (Dominant X Dominant). By contrasting the baseline model and the models with interaction effects, we obtained a *p*-value for each type of interaction effect. Source code for this analysis was developed on R (version 3.4.3).

**KEGG analysis.** The KEGG pathway enrichment analysis of the genes located on the genomic fragments showing strong epistasis was conducted by the Clusterprofiler package [15] in *R* program (version 3.4.3, 13). Pathways with FDR-corrected *P* values (*q-*value) < 0.05 were considered significantly overrepresented.

**Supplementary Results and Analyses**

**Variation of seed-related traits in S4 tetraploid populations relative to control populations.** To dissect the phenotypic effects of the multiple factors involved in the S4 tetraploid populations, we assessed the effect of each factor in separation, and factors in combination, on two seed-related traits, grain length and grain width, by comparing all the 12 relevant populations. These populations are: the diploid parents, Nipponbare and 93-11, reciprocal diploid F1 hybrids, N9 and 9N, autotetraploids of each parents, NPB-4X and 93-11-4X, immediate generation (S0) reciprocal allotetraploids, NN99-S0 and 99NN-S0, 1^st^ selfed generation (S1) reciprocal allotetraploids, NN99-S1 and 99NN-S1, and 4^th^-selfed generation (S4) reciprocal allotetraploids, NN99-S4 and 99NN-S4. For each trait, when compared with parents: (1) differences manifested in N9 and 9N are due to the effects of hybridization; (2) differences manifested in NPB-4X and 93-11-4X are due to the effects of polyploidization (genome doubling); (3) differences manifested in NN99-S0 and 99NN-S0 are due to the combined effects of hybridization and polyploidization (*i.e.*, allopolyploidization); (4) differences manifested in NN99-S1 and 99NN-S1 are due to the combined effects of allopolyploidization and sporadic HEs (only 1 meiosis involved); and (5) differences manifested in NN99-S4 and 99NN-S4 are due to the combined effect of allopolyploidization and many HEs (Fig. 2 and Fig. S8). We illustrated two seed-related traits, grain length and grain width (Fig. S4A), and compared both the mean values and standard deviations of each trait across the populations and tabulated the results (Fig. S4 B-D).

First, for mean values (Fig. S4 B and C), we found that: (1) the F1 hybrids are intermediates of parental values in both grain length and grain length, indicating hybridization did not generate heterotic effects on these two traits; (2) autotetraploidization of 93-11 significantly enlarged both traits, but that of Nipponbare did not, suggesting the effect of genome doubling on organ size might be both genotype- and organ-dependent; (3) the effects of allotetraploidization also showed genotype-specific effects for both traits, with NN99-S0/99NN-S0 surpassing only one parent; (4) the effects of combined allopolyploidization and sporadic HEs (comparing NN99-S1/99NN-S1 vs. NN99-S0/99NN-S0) on both traits are positive by large, but to variable degrees, being stronger for grain length than for grain width; (5) many HEs further strengthened the positive effects as evidenced by dramatically increased values in both traits in one crossing direction S4 tetraploids, i.e., 99NN (Fig. S4 B and C). Second, for the standard deviations (Fig. S4D), we found that although statistical differences emerged due to hybridization/genome doubling in separation or combination, and hence implicating their effects on within-population heterogeneity, their effects are almost negligible compared with that by HEs; this is evidenced by the fact that standard deviations of both NN99-S4 and 99NN-S4 populations that contain rampant HEs (Fig. 2 and Fig. S8) are significantly different from all the rest populations.

**HE rates per meiosis of S4 and S5 tetraploid populations.** Given the unexpected high number of HEs detected, we suspect that the HEs may have been not only cumulative over the generations, but also follow a “ratchet-like” fashion as proposed [16]. To test this possibility, we calculated HE rates of 2 consecutive selfed generation at population level. The S5 tetraploid population contained 45 euploid individuals, which are direct selfing progenies of random individuals of the 202 S4 tetraploids (Supplementary Fig. S1). In total 27,945 and 8,544 HEs were identified in the 202 S4 and 45 S5 tetraploids, respectively. The computational formula of HE rate per meiosis we used is: HE rate per meiosis = the number of HEs / (individual number * gamete number * meiosis times). In order to perform a statistical test, we divided each of the S4 and S5 populations into three subgroups that contained similar number of otherwise random individuals, and calculated the HE rates per meiosis for each subgroup separately. Results showed that the HE rate per meiosis in S5 population was significant higher than that in S4 population (19.0 ± 0.70 in S5 vs. 17.4 ± 0.46 in S4 per meiosis, or 0.79 ± 0.03 in S5 vs. 0.72 ± 0.02 in S4 per chromosome pair, *n* = 24, *P* = 0.0269, Student's t-test), lending support to the “polyploid-ratchet-like” metaphor [16].

**Allelic variations at sequence and expression levels of 4 prominent known-function genes, *GS3*, *TAC1*, *NAL1* and *DTH7*, between the two parents.** To explore the functional and/or expression variations of *GS3*, *TAC1*, *NAL1* and *DTH7* alleles between the two parents (NPB and 93-11) underlying their respective traits, we summarized the allelic variations of these four prominent known-function genes between the two parents at both sequence and expression levels (in the corresponding tissues) based on previous studies. *GS3* functions as a negative regulator of grain size and organ size, and relative to NPB, 93-11 had a C to A substitution at 165 bp downstream from the predicted translation start site (ATG), causing premature termination of the predicted protein and resulting in long grain [17]. Analysis of expression pattern of *GS3* gene showed that it was highly expressed in young panicle and gradually decreased with panicle development [17]. Both the expression levels and patterns of *GS3* were similar between NPB and 93-11, which suggests that the null phenotypic effect of *GS3* in 93-11 is due to its loss-of-function [17]. For *TAC1* gene, previous studies have reported that *TAC1* may serve as a member of a novel small gene family with specific functions in regulation of the tiller angle in *Graminae* plants [18, 19]. Only one single nucleotide polymorphism (SNP), a synonymous substitution, was detected in *TAC1* coding regions between NPB and 93-11 [18, 19]. Compared with 93-11 *TAC1* allele, a mutation from ‘AGGA’ to ‘GGGA’ in the 3’-splicing site of the 1.5-kb intron in 3’-UTR within *TAC1* locus decreases the expression level of *tac1* in the tiller bases, resulting in a compact plant architecture with a very small tiller angle in NPB [18, 19]. The *NAL1* gene encodes a putative trypsin-like serine/cysteine protease. The *NAL1* alleles of NPB and 93-11 had base differences at seven positions in the promoter region, including 3 single-base substitutions and 4 multi-base insertions and deletions; in the coding region, there were 1 and 2 single-base substitutions on the third and fifth exons, respectively, causing changes in the amino acids encoded [20, 21]. Regardless of the growth stage of heading or maturity, the relative expression level of *NAL1* in flag leaf was significantly higher in NPB than in 93-11 [20, 21]. These results suggest that both *NAL1* alleles in NPB and 93-11 are functional, but the 93-11 *NAL1* allele causes wider leaf than that of NPB [20, 21]. The *DTH7* gene contributes greatly to regulating rice photoperiodic flowering, plant architecture and grain productivity, and its function is largely affected by light conditions [22, 23]. In long days, *DTH7* acts downstream of the photoreceptor phytochrome B to repress the expression of *Ehd1*, an up-regulator of the “florigen” genes (*Hd3a* and *RFT1*), leading to delayed flowering. Compared to NPB *DTH7* coding region, 9 single-base substitutions and 1 eight-base insertions caused 8 amino acid changes and a frameshift mutation was also found in 93-11 *DTH7* allele [22, 23]. Comparison results of *DTH7* expression level showed that NPB *DTH7* allele had significantly higher expression than that of 93-11 in the leaf tissue.

**Background for epitasis and its four identification models.** Epistasis refers to the nonadditive interaction between non-allelic genes and can be divided into functional epistasis and statistical epistasis according to the detection methods and application scope [24-26]. Usually, functional epistasis applies to classical Mendelian genetics and molecular genetics and refers to genic interaction that one locus’ phenotypic effects mask the phenotypic effects of another locus, while statistical epistasis applies to evolutionary theory and quantitative genetics and refers to the non-additive interactions between non-allelic genes. For statistical epistasis, there are four identification models, additive by additive model (A by A), additive by dominant model (A by D), dominant by additive model (D by A) and dominant by dominant model (D by D). A by A model means both of the loci showed additive effect for a given trait when consider them separately; however, as a pair, the first locus shows opposite effects when the second locus at its alternative homozygous states, respectively, and an interdependent effect was evident that scales with homoeolog copy number; A by D model refers to the dominant effects of the second locus on a given trait are incrementally influenced by the first locus; D by A model is that the additive effects of the second locus on a given trait are dependent on a heterozygous state of the first locus; and D by D model means that the effect of the second locus on a given trait is contingent on the first locus being in a heterozygous state, and the two loci interacted more favorably when both loci were heterozygous.

**Pathway analysis for the fragments showing strong epistasis.** We have performed further KEGG analysis for genes located in the HE-related fragments that showed epistasis for each trait. We found that genes located on the genomic fragments showing strong digenic epistasis for five traits phenotyped (fertility, flag leaf length, grain width, thousand kernel weight and secondary branch number per panicle) were significantly enriched in the pathways that related to the corresponding traits (Dataset S5B). Taken flag-leaf length and grain width as examples, the pathways of photosynthesis (osa00195), ribosome (osa03010), oxidative phosphorylation (osa00190) and glutathione metabolism (osa00480) were enriched significantly in the genes located in the genomic fragment pairs showing digenic epistasis (all of the four epistasis models were involved, A by A, A by D, D by A and D by D) in flag-leaf length; the pathways of galactose metabolism (osa00052) and fructose and mannose metabolism (osa00051) were enriched repeatedly in each of the four epistasis models for grain width.

**Supplementary References**

1. Allen G, Flores-Vergara M and Krasynanski S. A modified protocol for rapid DNA isolation from plant tissues using cetyltrimethylammonium bromide. *Nat Protoc* 2006; **1:** 2320.

2. Li H and Durbin R. Fast and accurate short read alignment with Burrows-Wheeler transform. *Bioinformatics* 2009; **25:** 1754-1760.

3. Dobin A, Davis CA and Schlesinger F *et al.* STAR: ultrafast universal RNA-seq aligner. *Bioinformatics* 2013; **29:** 15-21.

4. International RGSP. The map-based sequence of the rice genome. *Nature* 2005; **436:** 793.

5. Der Auwera GA, Carneiro MO and Hartl C *et al.* From FastQ data to high-confidence variant calls: the genome analysis toolkit best practices pipeline. *Curr Protoc Bioinformatics* 2013; **43:** 11.10. 1-11.10. 33.

6. Zhang ZB, Fu TS and Liu ZJ *et al.* Extensive changes in gene expression and alternative splicing due to homoeologous exchange in rice segmental allopolyploids. *Theor Appl Genet* 2019; **132:** 2295-2308.

7. Xu CM, Bai Y and Lin XY *et al.* Genome-wide disruption of gene expression in allopolyploids but not hybrids of rice subspecies. *Mol Biol Evol* 2014; **31:** 1066-1076.

8. Li N, Xu CM and Zhang A *et al.* DNA methylation repatterning accompanying hybridization, whole genome doubling and homoeolog exchange in nascent segmental rice allotetraploids. *New Phytol* 2019; **223:** 979-992*.*

9. Li H, Handsaker B and Wysoker A *et al.* The sequence alignment/map format and SAMtools. *Bioinformatics* 2009; **25:** 2078-2079.

10. Forsythe ES, Sharbrough J and Havird JC. CyMIRA: The Cytonuclear Molecular Interactions Reference for *Arabidopsis*. *Genome Biol Evol* 2019; **11:** 2194-2202.

11. Armenteros JA, Salvatore M and Emanuelsson O *et al.* Detecting sequence signals in targeting peptides using deep learning. *Life Sci* 2019; **2:** e201900429.

12. Sperschneider J, Catanzariti A and Deboer KD *et al.* LOCALIZER: subcellular localization prediction of both plant and effector proteins in the plant cell. *Sci Rep* 2017; **7:** 1-14.

13. Liu X, Huang M and Fan B *et al.* Iterative usage of fixed and random effect models for powerful and efficient genome-wide association studies. *PLoS Genet* 2016; **12:** e1005767.

14. Zeng Z-B, Wang T and Zou W, Modeling quantitative trait loci and interpretation of models. *Genetics* 2005; **169:** 1711-1725.

15. Yu G, Wang L and Han Y *et al*. ClusterProfiler: an R package for comparing biological themes among gene clusters. *OMICS* 2012; **16:** 284-287.

16. Gaeta RT and Pires JC. Homoeologous recombination in allopolyploids: The polyploid ratchet. *New Phytol* 2010; **186:** 18-28.

17. Mao H, Sun S and Yao J *et al.* Linking differential domain functions of the GS3 protein to natural variation of grain size in rice. *Proc Natl Acad Sci USA* 2010; **107:** 19579-19584.

18. Jiang J, Tan L and Zhu Z *et al.,* Molecular evolution of the *TAC1* gene from rice (*Oryza sativa* L.). *J Genet Genomics* 2012; **39:** 551-560.

19. Yu B, Lin Z and Li H *et al.* *TAC1*, a major quantitative trait locus controlling tiller angle in rice. *Plant J* 2007; **52:** 891-898.

20. Qi J, Qian Q and Bu Q *et al.* Mutation of the rice *Narrow leaf1* gene, which encodes a novel protein, affects vein patterning and polar auxin transport. *Plant Physiol* 2008; **147:** 1947-1959.

21. Zhang G, Li S and Wang L *et al.* *LSCHL4* from japonica cultivar, which is allelic to *NAL1*, increases yield of indica super rice 93-11. *Mol Plant* 2014; **7:** 1350-1364.

22. Gao H, Jin M and Zheng X *et al.* *Days to heading 7*, a major quantitative locus determining photoperiod sensitivity and regional adaptation in rice. *Proc Natl Acad Sci USA* 2014; **111:** 16337-16342.

23. Koo B, Yoo S and Park J *et al.* Natural Variation in *OsPRR37* Regulates Heading Date and Contributes to Rice Cultivation at a Wide Range of Latitudes. *Mol Plant* 2013; **6:** 1877-1888.

24. Khan A, Dinh DM and Schneider D *et al.* Negative epistasis between beneficial mutations in an evolving bacterial population. *Science* 2011; **332:** 1193-1196.

25. Phillips PC. Epistasis-the essential role of gene interactions in the structure and evolution of genetic systems. *Nat Rev Genet* 2008; **9:** 855-867.

26. Sanjuán R and Elena S. Epistasis correlates to genomic complexity. *Proc Natl Acad Sci USA* 2006; **103:** 14402-14405.

27. Liu XY, Sun S and Wu Y *et al.* Dual‐color oligo‐FISH can reveal chromosomal variations and evolution in *Oryza* species. *Plant J* 2020; **101,** 112-121.


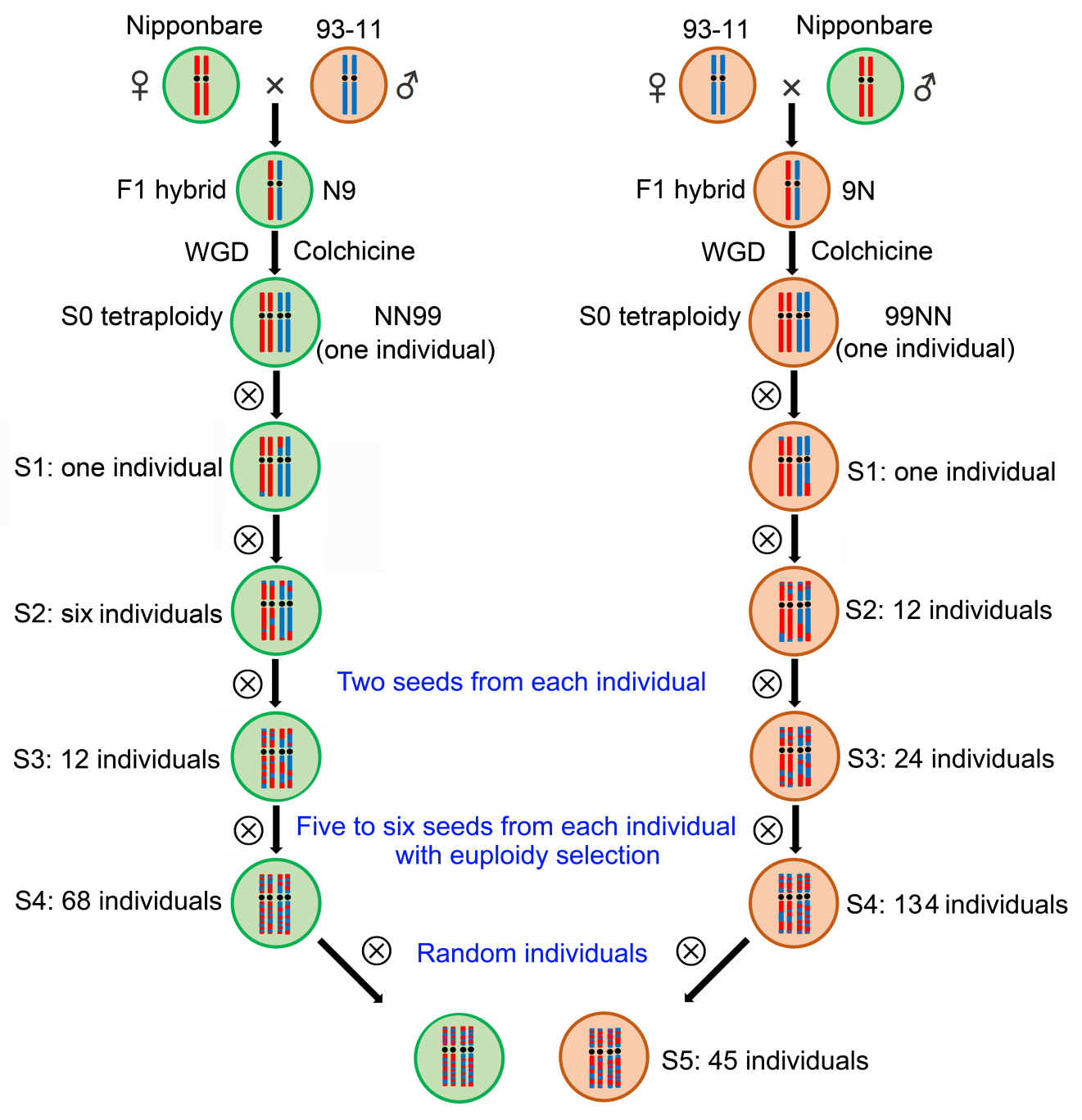


**Fig. S1.** Diagrammatic illustration of the plant system used in this study. Reciprocal F1 hybrids were produced by crossing two subspecies, *japonica* and *indica*, of rice (*Oryza sativa* L.), followed by colchicine-mediated chromosome doubling. Doubled plants (S0) were selfed for four successive generations. The number of individuals used at each generation are denoted. Hypothetical chromosome constitutions for each tetraploid individual are depicted. Different colors of the hypothetic diagrammed chromosomes and cytoplasm (maternal inheritance) refer to their parental origins.


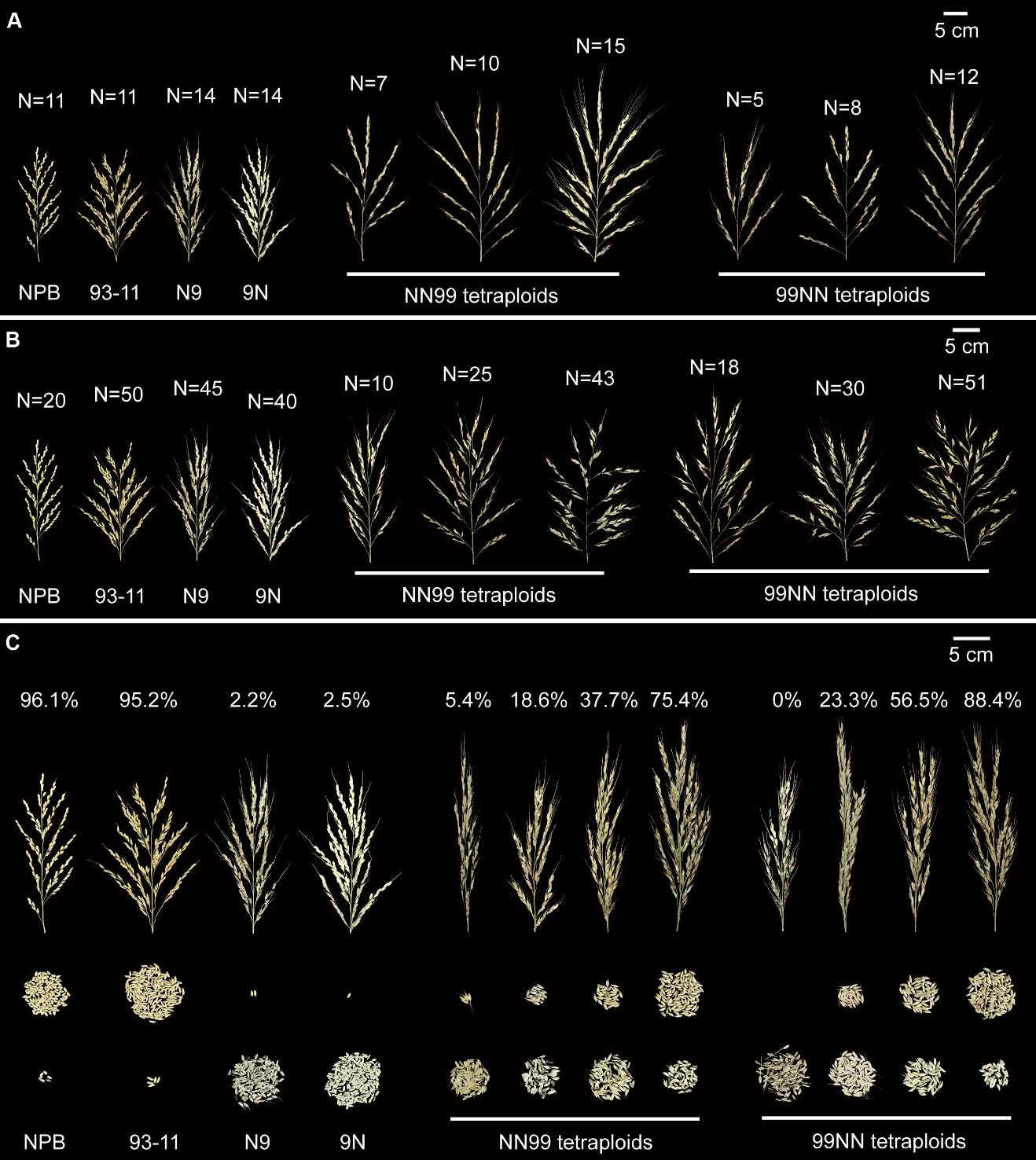


**Fig. S2.** Illustration of panicle-related traits of the diploid parents, Nipponbare (NPB) and 93-11, reciprocal F1 hybrids (N9 and 9N) and reciprocal S4 tetraploids (NN99 and 99NN). (A) and (B) show primary and secondary branch numbers per panicle, respectively. (C) shows to panicle fertility; panels from top to bottom showing a given panicle, its filled and empty grains, respectively.


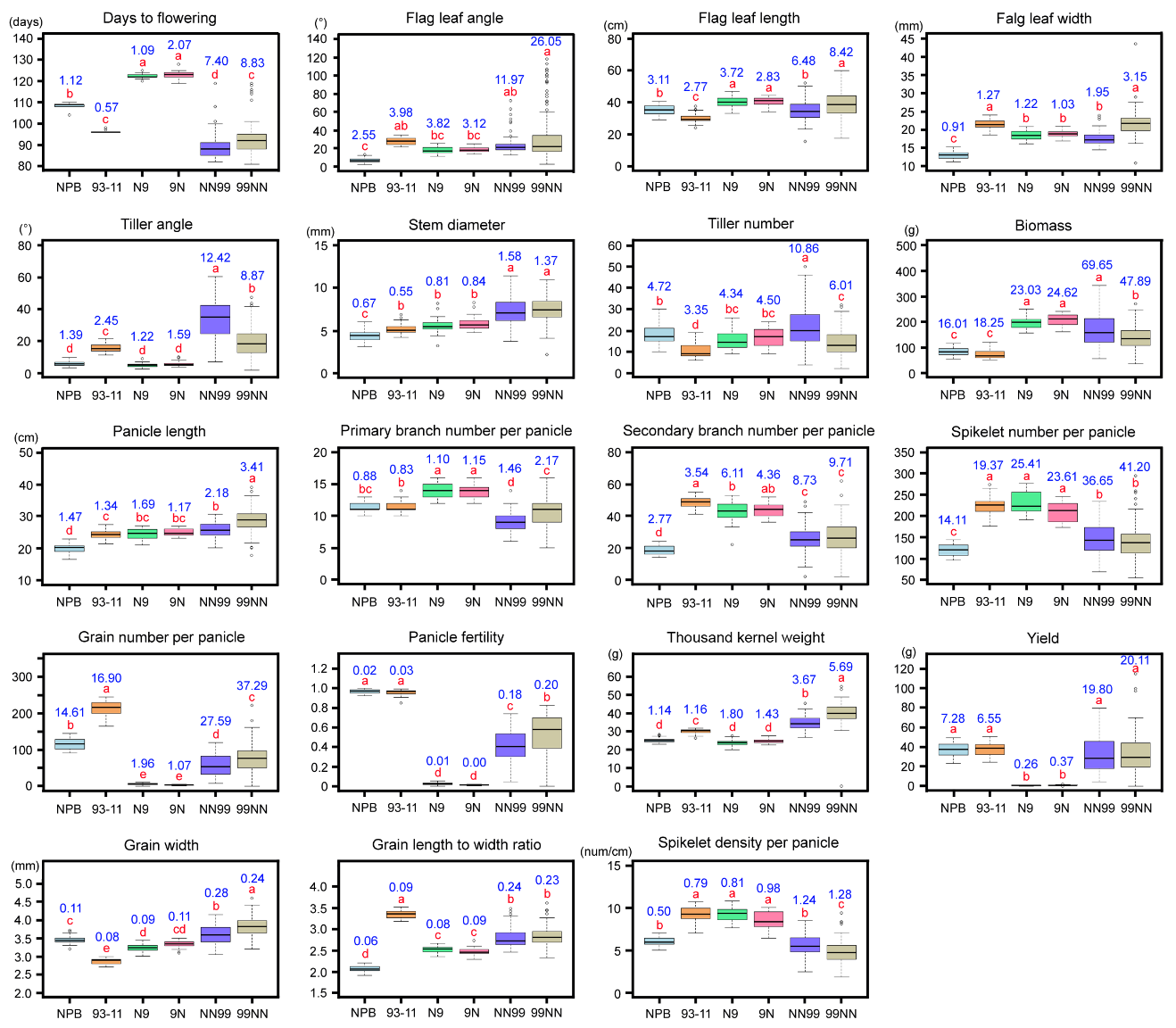


**Fig. S3.** Quantification of 19 phenotypic traits of the diploid parents (Nipponbare/NPB and 93-11), reciprocal F1 hybrids (N9 and 9N) and S4 tetraploids in both NN99 and 99NN directions by boxplots. For each panel, letters above each box represent statistically different phenotypic distributions in each comparison, and the blue numbers above each box represent the relevant standard deviations of the data from each box.


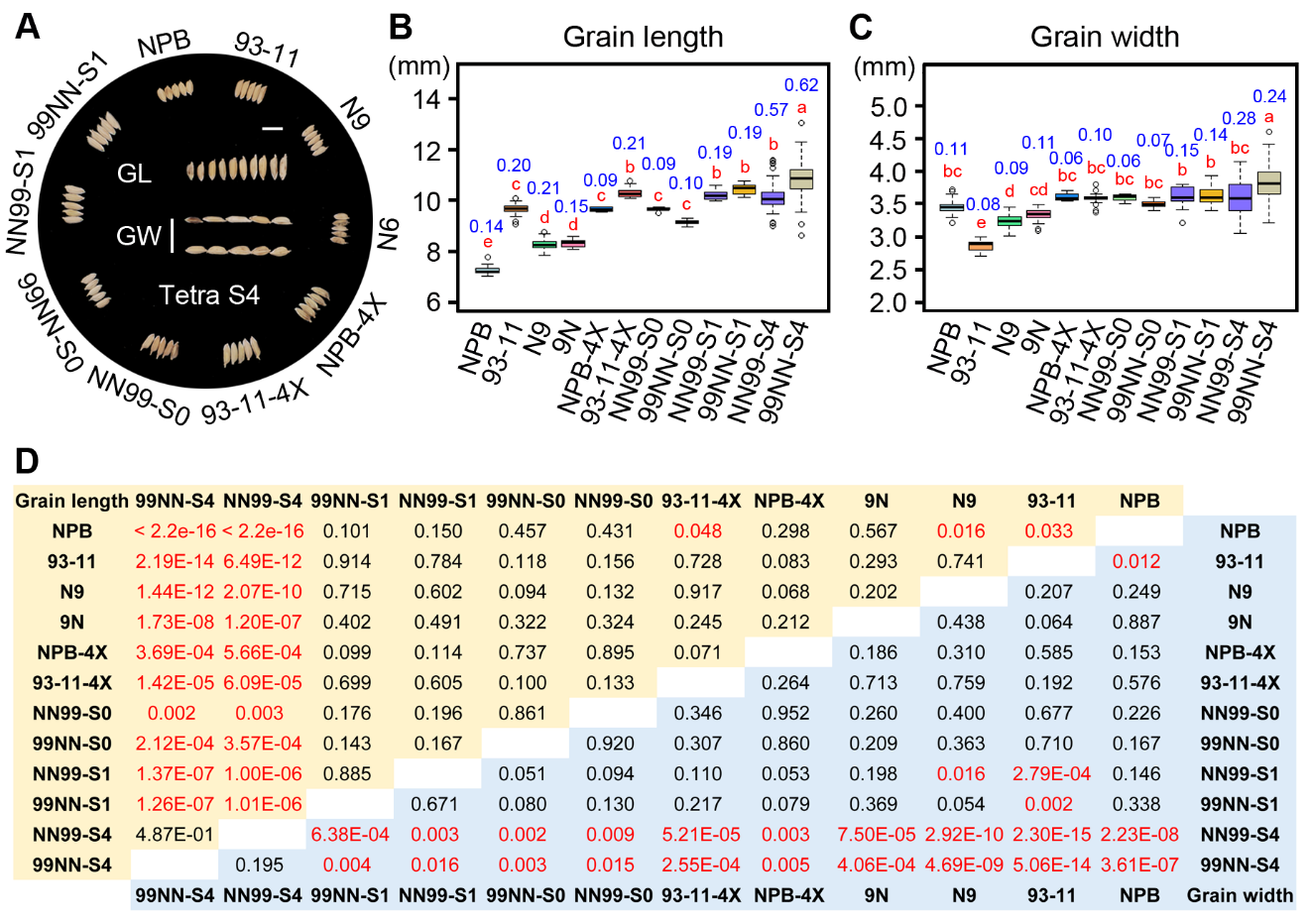


**Fig. S4.** Illustration and quantification of grain length and grain width of 12 populations, including the two diploid parents, Nipponbare (NPB) and 93-11, reciprocal F1 hybrids, N9 and 9N, autotetraploids of parents, NPB-4X and 93-11-4X and S0, S1 and S4 tetraploids in both crossing directions (NN99 and 99NN), and comparisons of standard deviations across all tested populations. (A) Illustration of variations in grain length and grain width of the foregoing rice populations, bar = 1 cm. Seeds illustrating variations in grain length (GL) and grain width (GW) of the S4 tetraploids (Tetra S4; did not show reciprocals here because they are not different in this aspect) are from different lines. (B) and (C) Quantification of grain length and grain width by boxplots, respectively; letters above each box denote statistical differences in the phenotypic distributions in each comparison; blue decimal above each box refers to the relevant standard deviations of data in the respective box. (D) Statistical test (F test) of pairwise comparisons of standard deviations across the 12 populations for grain length (above diagonal) and grain width (below diagonal).


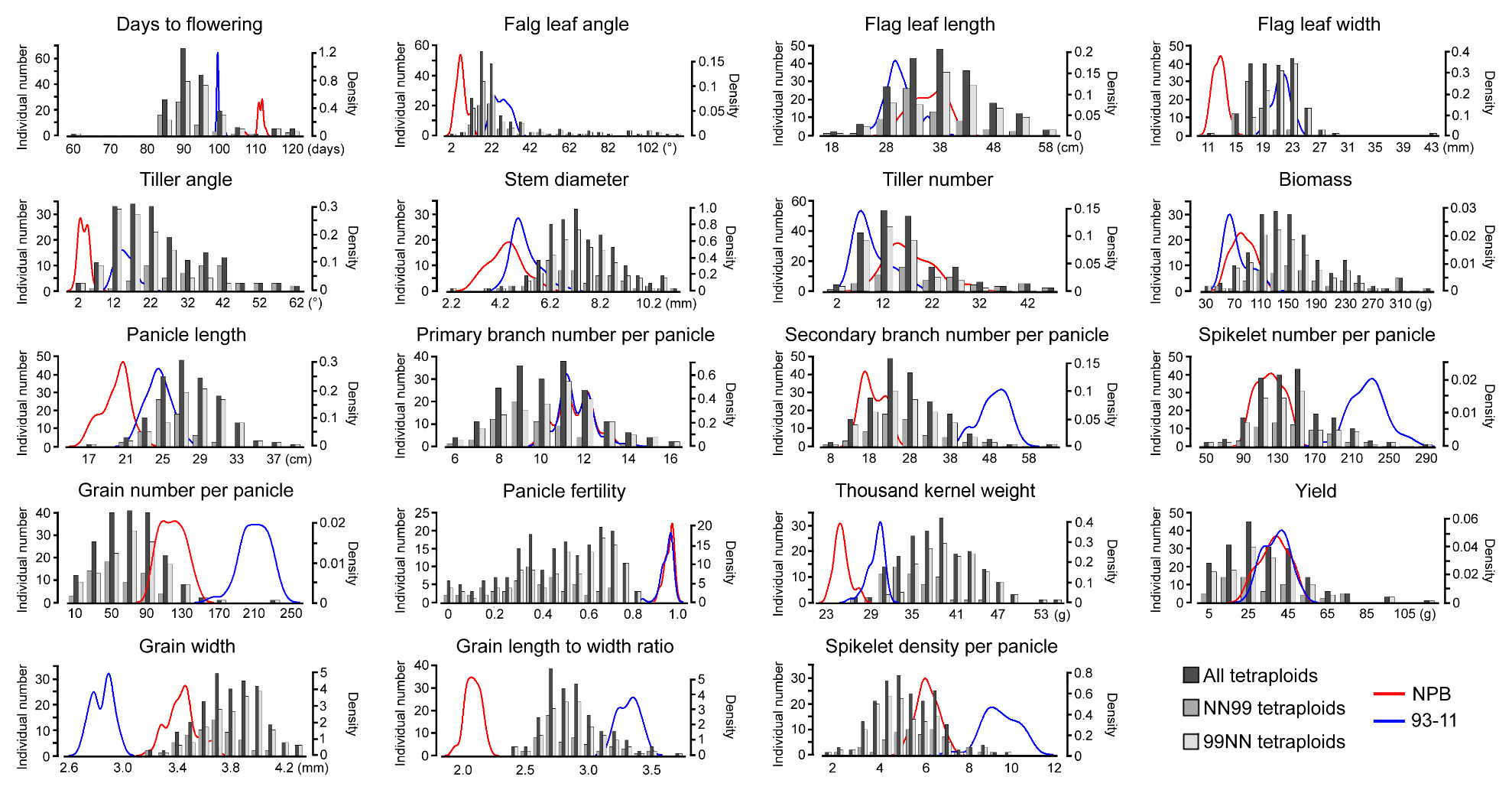
 **Fig. S5.** Quantification of 19 phenotypic traits of the diploid parents (NPB and 93-11) and S4 tetraploids in both NN99 and 99NN directions by density plot and histograms, respectively. The left and right ordinates are for the histograms and density plots (red and blue curves), respectively.


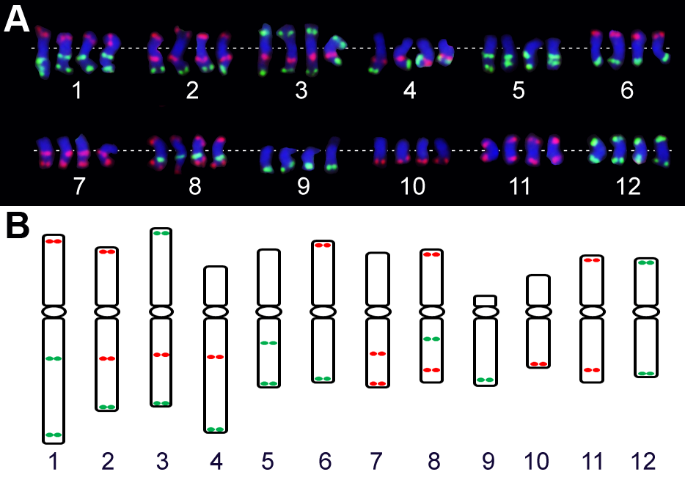


**Fig. S6.** Identification of rice euploid tetraploids by dual-color oligo-Fluorescence in situ hybridization (FISH) analysis of root-tip mitotic metaphase chromosomes [27]. (A) Caryogram of the 12 rice chromosomes of a typical euploid tetraploid. Green and red signals are from FAM-green and Texas-red probes, respectively. (B) depicted ideogram based on (A).


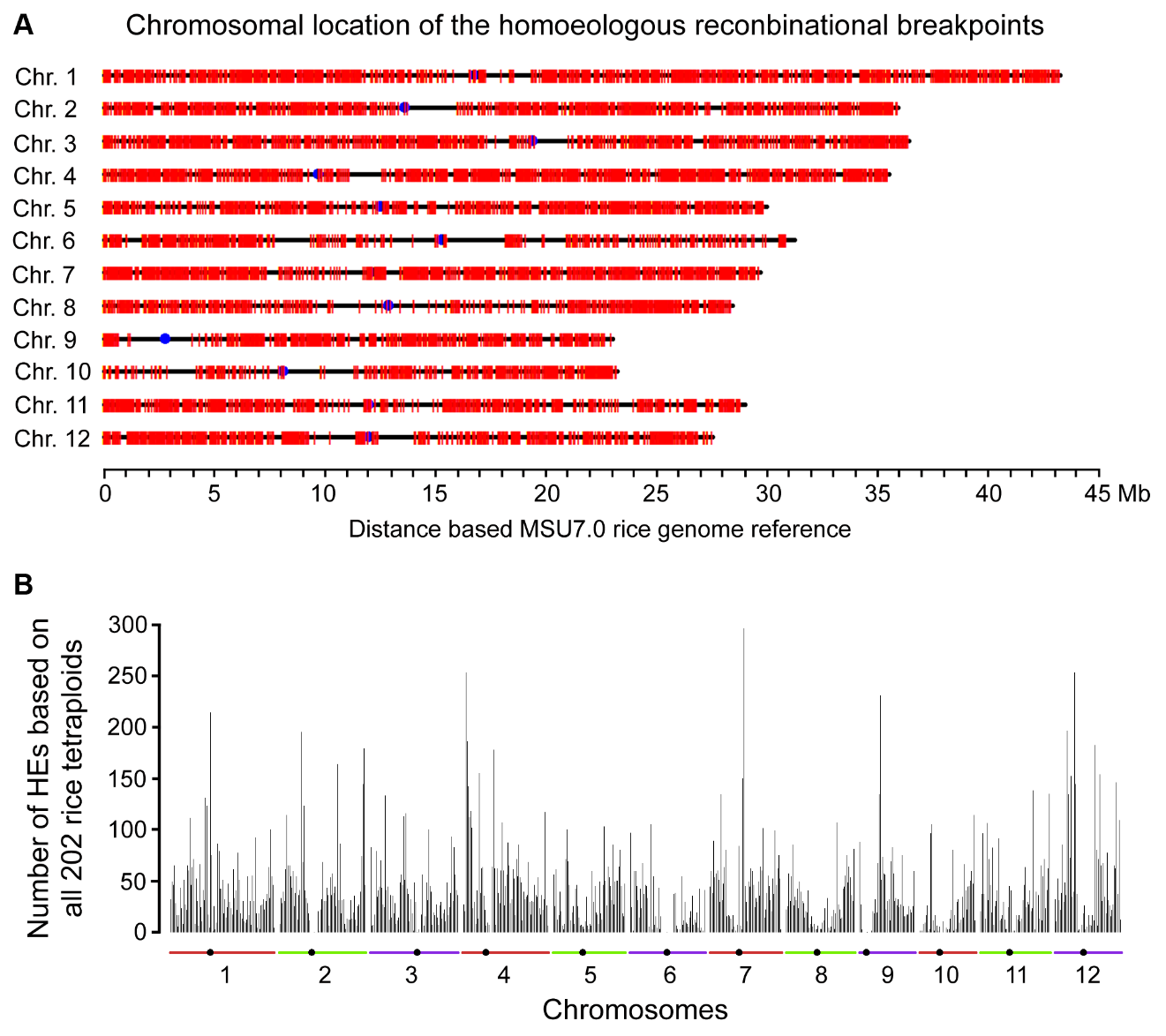


**Fig. S7.** Plots showing quantification of all identified HEs for each chromosome aggregated across the 202 re-sequenced individuals. (A) Chromosomal location of the homoeologous recombination breakpoints based on 202 S4 tetraploid individuals. Each red vertical bar represents a breakpoint, the thick black lines and blue dots represent chromosomes and centromeres, respectively. (B) Histogram showing the number of homoeologous recombination breakpoints aggregated across the 202 re-sequenced individuals among 12 rice chromosomes and within one given chromosome.


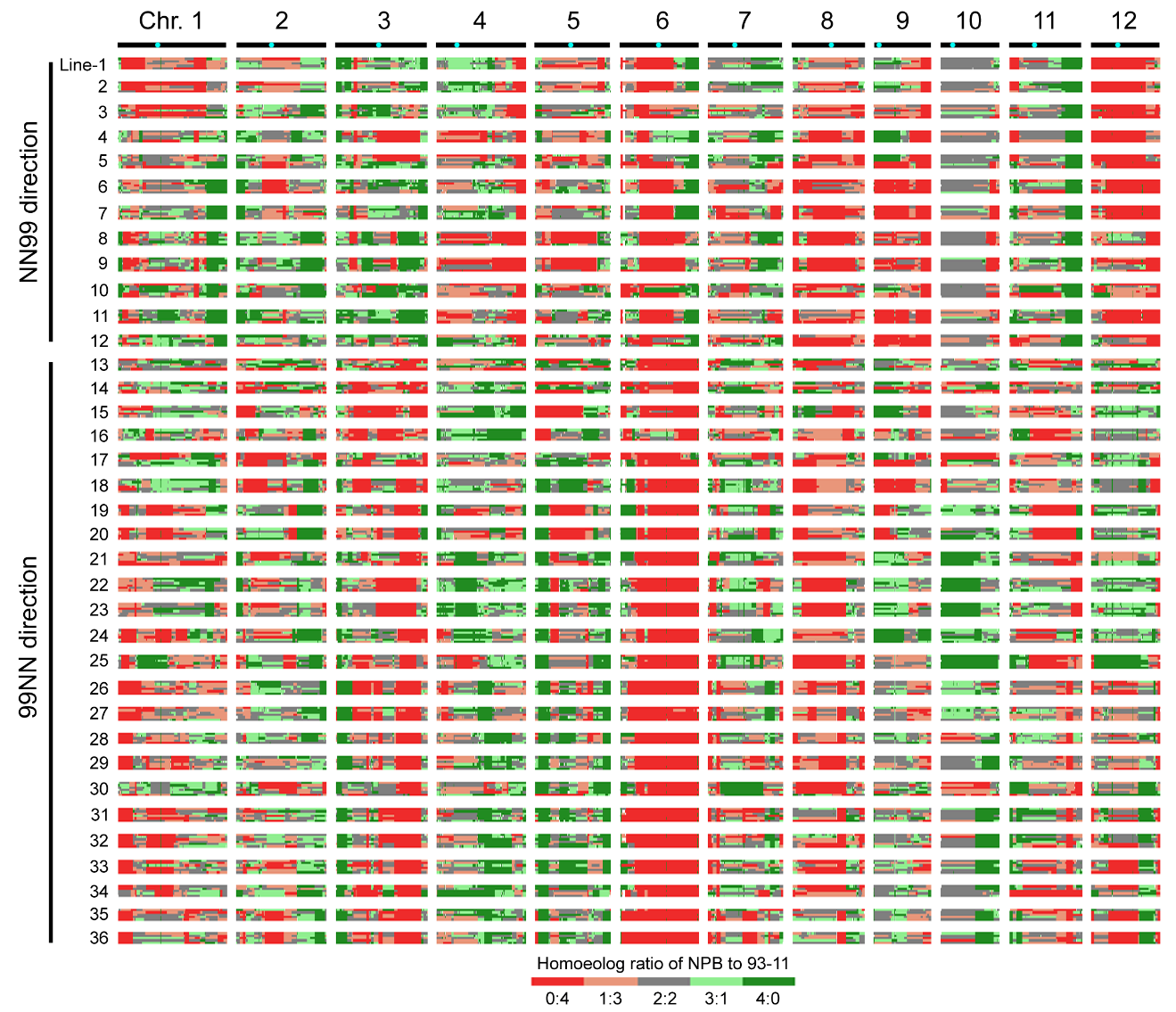


**Fig. S8** Heatmaps depicting the genomic landscape of all of 202 re-sequenced S4 euploid tetraploid individuals from 36 selfed lines of reciprocal cross directions. Different colors show the different homoeolog compositions, e.g., orange represents the homoeologous ratio between NPB and 93-11 in a given locus is 1:3. Each row represents one tetraploid individual, the 12 columns represent the 12 chromosomes in rice genome, and the black dots on the chromosomal pattern diagrams represent centromeres. The cross direction and line names are labeled on the left of the rows.


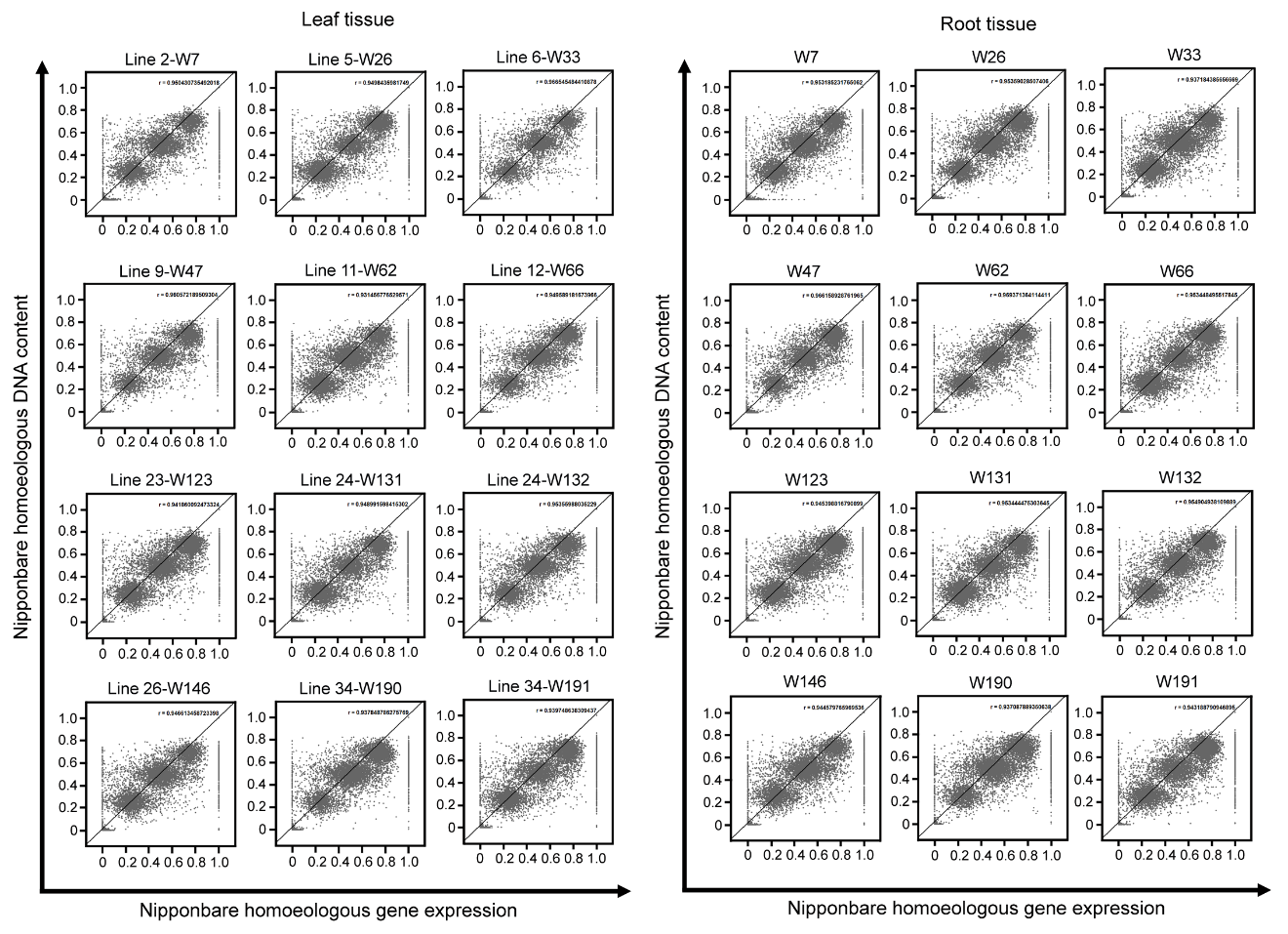


**Fig. S9.** DNA-dependent homoeologous gene expression in leaf (left) and root (right) tissues of 12 S4 euploid tetraploid individuals. Each gene is represented as a dot in the scatter plots, in which the *x*- and *y*-axis coordinates of each dot correspond to the relative NIP homoeolog expression level and DNA copy number, respectively. The dotted diagonal line in each plot depicts the regression line of all the dots.


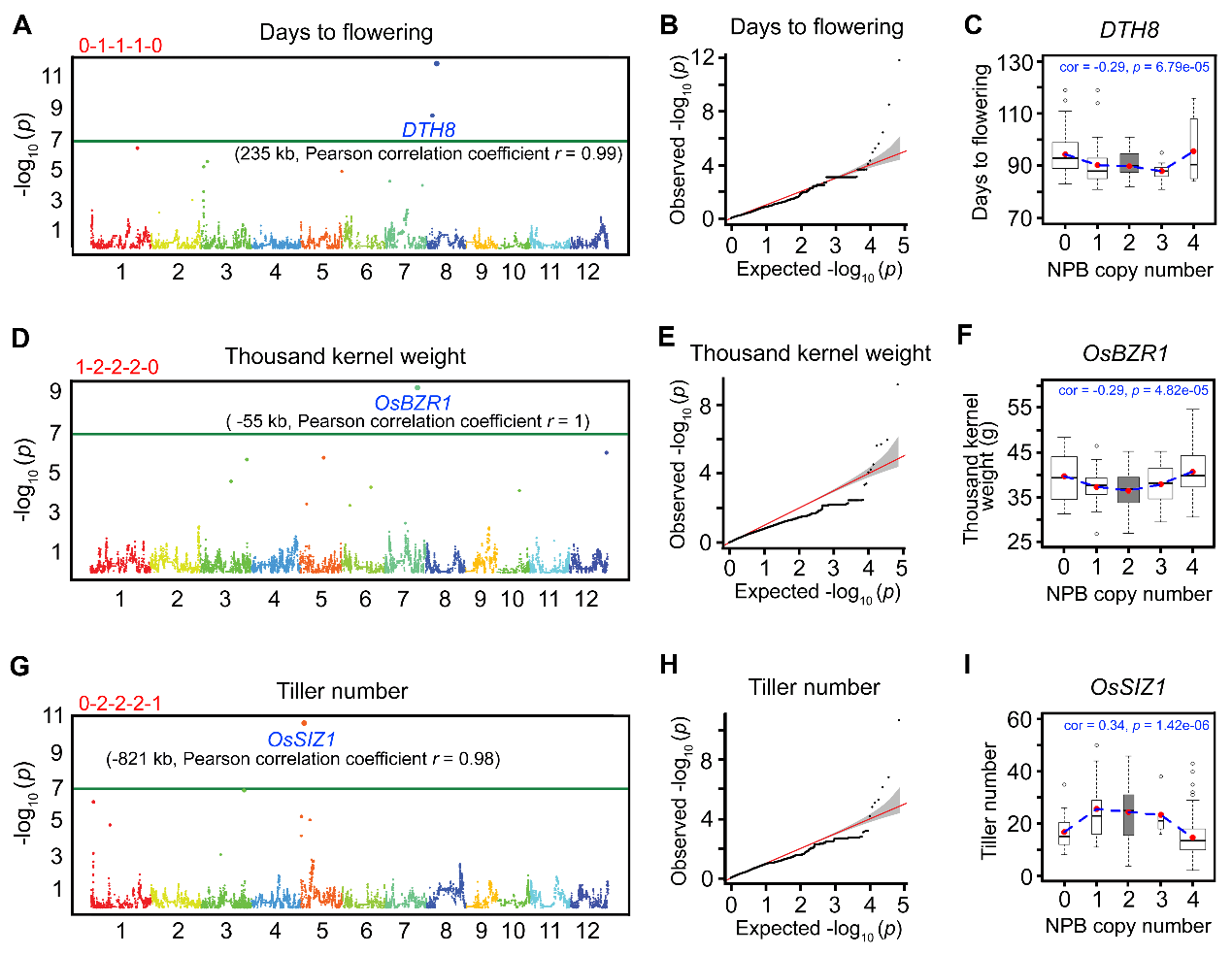


**Fig. S10.** GWAS of days to flowering, thousand kernel weight and tiller number with three dominant models by FarmCPU R scripts. (A), (D) and (G) are Manhattan plots, wherein the green lines represent thresholds based on Bonferroni test, with adjacent genes controlling the given trait labeled in blue font below the corresponding identified locus. (B), (E) and (H) are Quantile-Quantile plots of p-value. (C), (F) and (I) are boxplots showing dominance between phenotype and NPB copy number in each of the three genes.


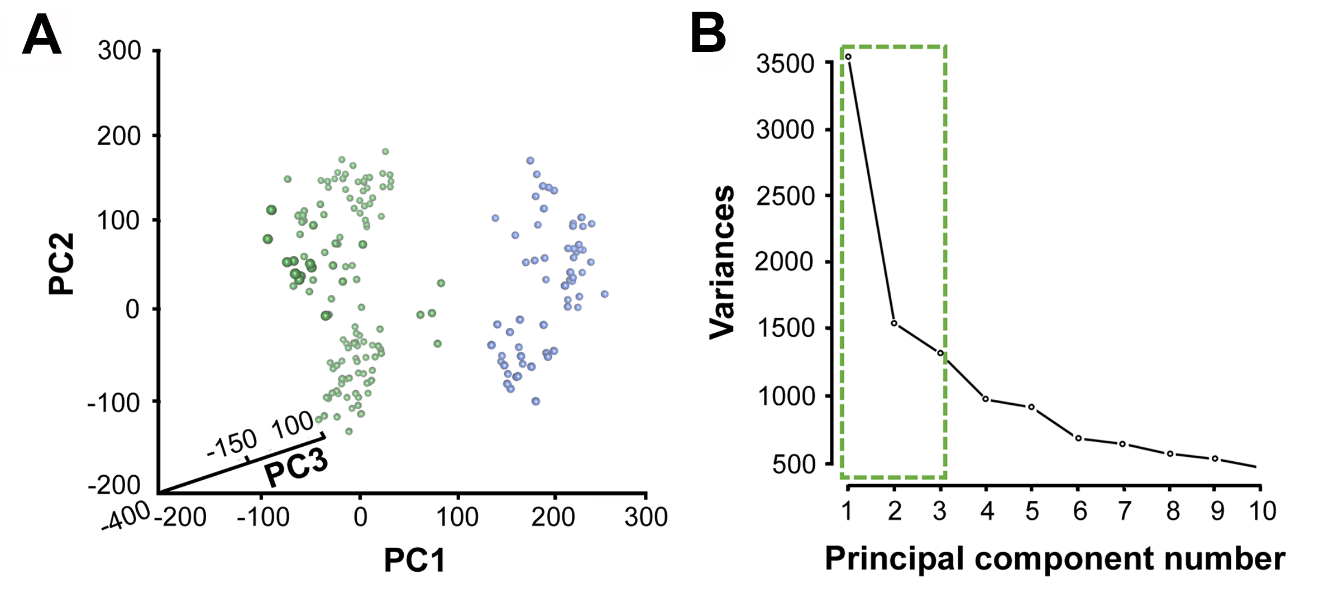


**Fig. S11.** (A) Population structure and (B) The contribution of each principal component to phenotypic variance.

**Table S1.** The number and percentage of individuals with transgressive phenotypic values in the tetraploid population for each trait.

| **Traits** | **Total transgressive tetraploid number** | | | |
| --- | --- | --- | --- | --- |
|  | **Over-transgressivity** | **Under-transgressivity** | **All** | **Significant difference test** |
| **Days to flowering** | 11 (6.11%) | 143 (79.44%) | 154 (85.56%) | < 2.2e-16 |
| **Plant height** | 28 (14.36%) | 124 (63.59%) | 152 (77.95%) | 1.3e-15 |
| **Flag leaf length** | 89 (45.64%) | 18 (9.23%) | 107 (54.87%) | 1.8e-12 |
| **Flag leaf width** | 48 (24.62%) | 1 (0.51%) | 49 (25.13%) | 1.8e-13 |
| **Flag leaf angle** | 47 (24.23%) | 1 (0.52%) | 48 (24.74%) | 3.5e-13 |
| **Tiller angle** | 125 (64.1%) | 3 (1.54%) | 128 (65.64%) | < 2.2e-16 |
| **Stem diameter** | 178 (91.28%) | 2 (1.03%) | 180 (92.31%) | < 2.2e-16 |
| **Tiller number** | 117 (60%) | 17 (8.72%) | 134 (68.72%) | < 2.2e-16 |
| **Panicle length** | 144 (74.23%) | 1 (0.52%) | 145 (74.74%) | < 2.2e-16 |
| **Primary branch number per panicle** | 24 (12.44%) | 106 (54.92%) | 130 (67.36%) | 1.7e-13 |
| **Secondary branch number per panicle** | 2 (1.04%) | 20 (10.36%) | 22 (11.4%) | 1.2e-4 |
| **Spikelet number per panicle** | 3 (1.55%) | 31 (16.06%) | 34 (17.62%) | 7.7e-07 |
| **Grain number per panicle** | 0 (0%) | 159 (82.38%) | 159 (82.38%) | < 2.2e-16 |
| **Spikelet density per panicle** | 0 (0%) | 123 (63.73%) | 123 (63.73%) | < 2.2e-16 |
| **Fertility** | 0 (0%) | 193 (100%) | 193 (100%) | < 2.2e-16 |
| **Thousand kernel weight** | 173 (93.01%) | 0 (0%) | 173 (93.01%) | < 2.2e-16 |
| **Yield** | 47 (25.27%) | 97 (52.15%) | 144 (77.42%) | 3.8e-05 |
| **Grain length** | 167 (89.3%) | 0 (0%) | 167 (89.3%) | < 2.2e-16 |
| **Grain width** | 138 (73.8%) | 0 (0%) | 138 (73.8%) | < 2.2e-16 |
| **Grain length to width ratio** | 3 (1.6%) | 0 (0%) | 3 (1.6%) | 0.3 |
| **Biomass** | 159 (84.13%) | 3 (1.59%) | 162 (85.71%) | < 2.2e-16 |

**Table S2.** Homoeologous recombination number and HE frequency on each of the 12 rice chromosomes of six randomly selected S4 euploid tetraploid individuals and of all 202 re-sequenced tetraploid individuals.

| **Chromosome** | **Chr. size (Mb)** | **HE number** | | | | | | | **HE frequency (cM/Mb)** |
| --- | --- | --- | --- | --- | --- | --- | --- | --- | --- |
|  |  | **W71** | **W73** | **W78** | **W97** | **W149** | **W151** | **Mean of 202 tetraploids** |  |
| 1 | 41.3 | 20 | 8 | 12 | 13 | 12 | 10 | **16.81** | 20.35 |
| 2 | 34.3 | 13 | 12 | 11 | 14 | 6 | 12 | 14.47 | 21.09 |
| 3 | 34.7 | 16 | 10 | 12 | 11 | 10 | 10 | 12.61 | 18.17 |
| 4 | 33.9 | 21 | 6 | 6 | 9 | 9 | 11 | **18.25** | 26.92 |
| 5 | 28.6 | 11 | 10 | 7 | 9 | 11 | 20 | 9.97 | 17.43 |
| 6 | 29.8 | 3 | 5 | 8 | 5 | 4 | 4 | **6.41** | **10.76** |
| 7 | 28.3 | 9 | 10 | 7 | 27 | 13 | 16 | 14.12 | 24.95 |
| 8 | 27.1 | 7 | 7 | 8 | 6 | 6 | 8 | **7.82** | 14.43 |
| 9 | 22 | 11 | 5 | 4 | 5 | 6 | 5 | 8.25 | 18.75 |
| 10 | 22.1 | 5 | 5 | 8 | 7 | 7 | 2 | **5.38** | 12.17 |
| 11 | 27.7 | 5 | 5 | 12 | 10 | 11 | 8 | 9.95 | 17.96 |
| 12 | 26.3 | 4 | 6 | 11 | 9 | 13 | 14 | **14.33** | **27.24** |
| Mean | - | 10 | 7 | 9 | 10 | 9 | 10 | 11.53 | 19.18 |
| Upper threshold | | | | | | | | 17 | 27 |
| Lower threshold | | | | | | | | 6 | 12 |

**Table S3.** The number of homoeologous recombination breakpoints aggregated across the 202 re-sequenced individuals in each 500 kb genomic bin of pericentromeric regions, subtelomeric regions and whole-chromosomes. Pericentromeric regions were defined as three consecutive 500 kb bins harboring a centromere, and subtelomeric regions were defined as four consecutive 500 kb bins from the end of each chromosomal arm.

| **Chromosome** | **Whole-chromosome regions** | **Subtelomeric region** | **Pericentromeric region** | ***p* by T-test, Tele. vs. whole-chromosome** | ***p* by T-test, Cent. vs. whole-chromosome** |
| --- | --- | --- | --- | --- | --- |
| **1** | 39.02 ± 33.67 | 49.5 ± 27.29 | 97.67 ± 106.82 | 0.4 | 0.01 |
| **2** | 43.61 ± 40.31 | 68.88 ± 60.8 | 0 ± 0 | 0.12 | 0.07 |
| **3** | 35.38 ± 28.53 | 41.13 ± 31.02 | 0.67 ± 1.15 | 0.59 | 0.04 |
| **4** | 53.42 ± 46.49 | 83.38 ± 92.23 | 12 ± 7.94 | 0.13 | 0.13 |
| **5** | 33.55 ± 25.39 | 33.14 ± 20.46 | 5 ± 2 | 0.89 | 0.06 |
| **6** | 21.57 ± 24.3 | 28.5 ± 35.19 | 0 ± 0 | 0.48 | 0.13 |
| **7** | 48.34 ± 44.99 | 52.63 ± 26.2 | 32.33 ± 44.75 | 0.79 | 0.55 |
| **8** | 28.71 ± 23.56 | 41.75 ± 20.28 | 3.67 ± 2.08 | 0.14 | 0.07 |
| **9** | 35.45 ± 40.89 | 30.25 ± 29.54 | 0 ± 0 | 0.73 | 0.23 |
| **10** | 24.7 ± 29.52 | 30.13 ± 40.73 | 2 ± 2.65 | 0.65 | 0.19 |
| **11** | 35.88 ± 31.73 | 56.38 ± 40.01 | 36.33 ± 10.97 | 0.1 | 0.98 |
| **12** | 52.64 ± 56.5 | 53.63 ± 48.54 | 17 ± 9.54 | 0.96 | 0.28 |
| **Average** | 38.23 ± 37.74 | 47.58 ± 44.31 | 17.22 ± 39.23 | 0.03 | 0 |

**Table S4.** Average percentages of the five homoeolog composition types, homozygous loci and heterozygous loci on each chromosome, and symmetry analysis between homoeolog composition types of 4:0 and 0:4 and between 3:1 and 1:3.

| **Genomic type** | **1** | **2** | **3** | **4** | **5** | **6** | **7** | **8** | **9** | **10** | **11** | **12** | **Mean value** |
| --- | --- | --- | --- | --- | --- | --- | --- | --- | --- | --- | --- | --- | --- |
| **N : 9 = 4 : 0** | 13.93% | 20.39% | 20.33% | 24.56% | 30.14% | 10.70% | 18.23% | 3.75% | 12.75% | 20.32% | 18.63% | 15.58% | 17.44% |
| **N : 9 = 3 : 1** | 15.20% | 20.68% | 11.43% | 17.67% | 9.82% | 4.53% | 16.89% | 6.67% | 13.93% | 15.39% | 11.48% | 12.30% | 13.00% |
| **N : 9 = 2 : 2** | 25.96% | 26.06% | 19.93% | 23.88% | 25.71% | 8.27% | 28.10% | 18.92% | 26.18% | 43.82% | 27.44% | 23.61% | 24.82% |
| **N : 9 = 1 : 3** | 20.55% | 16.97% | 13.59% | 18.23% | 15.02% | 6.54% | 17.03% | 24.37% | 16.70% | 11.73% | 19.74% | 14.01% | 16.21% |
| **N : 9 = 0 : 4** | 24.36% | 15.90% | 34.73% | 15.66% | 19.30% | 69.96% | 19.74% | 46.28% | 30.43% | 8.74% | 22.72% | 34.50% | 28.53% |
| **Homozygosity** | 38.29% | 36.29% | 55.05% | 40.22% | 49.45% | 80.66% | 37.98% | 50.03% | 43.19% | 29.06% | 41.35% | 50.09% | 45.97% |
| **Heterozygosity** | 61.71% | 63.71% | 44.95% | 59.78% | 50.55% | 19.34% | 62.02% | 49.97% | 56.81% | 70.94% | 58.65% | 49.91% | 54.03% |
| **Symmetry between 4:0 and 0:4** | < 2.2e-16 | 3.6E-10 | < 2.2e-16 | < 2.2e-16 | < 2.2e-16 | < 2.2e-16 | < 2.2e-16 | < 2.2e-16 | < 2.2e-16 | < 2.2e-16 | 1.4E-06 | < 2.2e-16 | < 2.2e-16 |
| **Symmetry between 3:1 and 1:3** | < 2.2e-16 | 3.5E-07 | 2.0E-04 | 0.37 | 3.9E-16 | 1.8E-06 | 0.86 | < 2.2e-16 | 7.3E-04 | < 2.2e-16 | < 2.2e-16 | 1.0E-02 | 3.4E-06 |

**Table S5.** Reproduction-related traits in the 5% plants that did not harbor homo-cytonuclear compositions and their respective siblings of the 95%.

| **Traits** | **5% plants** | **95% plants** | ***P* by Student's t-test** |
| --- | --- | --- | --- |
| **Fertility** | 36.43% ± 2.14% | 76.00% ± 1.37% | 1.13E-05 |
| **Grain number per panicle** | 52.33 ± 18.77 | 107.33 ± 8.14 | 9.62E-03 |

**Table S6.** Cytonuclear gene numbers for the three featured genomic groups and whole rice genome.

| **Group category** | **Numbers of cytonuclear genes** | **Size of genomic region (Mb)** | **Numbers of cytonuclear genes per Mb** | ***P* by Pearson's Chi-squared test compared with whole rice genome** |
| --- | --- | --- | --- | --- |
| **Group I** | 32 | 9.78 | 3.27 | 1.02E-04 |
| **Group II** | 36 | 13.73 | 2.62 | 5.74E-03 |
| **Group III** | 79 | 21.95 | 3.60 | 1.32E-11 |
| **Three groups together** | 147 | 45.46 | 3.23 | 3.32E-14 |
| **Whole rice genome** | 672 | 400 | 1.68 | - |

**Table S7.** Number and percentage of homoeolog copy number-correlated homoeologous expression genes in 12 randomly chosen tetraploid individuals.

| **Tissue** | **Category** | **W1** | **W4** | **W9** | **W10** | **W11** | **W12** | **W13** | **W15** | **W16** | **W17** | **W18** | **W19** |
| --- | --- | --- | --- | --- | --- | --- | --- | --- | --- | --- | --- | --- | --- |
| **Leaf** | **Number of expressed genes** | 12256 | 12139 | 11761 | 13332 | 12547 | 13291 | 13800 | 13745 | 12694 | 12854 | 12781 | 13103 |
|  | **Number of DNA-correlated genes** | 10976 | 11040 | 10463 | 11614 | 11438 | 11689 | 11931 | 12098 | 10996 | 11249 | 11270 | 11686 |
|  | **Percentage of DNA-correlated genes** | 89.56% | 90.95% | 88.96% | 87.11% | 91.16% | 87.95% | 86.46% | 88.02% | 86.62% | 87.51% | 88.18% | 89.19% |
| **Root** | **Number of expressed genes** | 14466 | 14265 | 15134 | 15095 | 15399 | 15511 | 15050 | 14965 | 13892 | 14808 | 14883 | 14672 |
|  | **Number of DNA-correlated genes** | 13009 | 13079 | 13441 | 13315 | 14146 | 13793 | 13155 | 13200 | 12141 | 12960 | 13290 | 13100 |
|  | **Percentage of DNA-correlated genes** | 89.93% | 91.69% | 88.81% | 88.21% | 91.86% | 88.92% | 87.41% | 88.21% | 87.40% | 87.52% | 89.30% | 89.29% |

**Table S8.** Known genes adjacent to identified loci in GWAS that are causal to the phenotypic divergence between Nipponbare and 93-11.

| **Traits** | **Model** | **Fragment** | **Chr.** | **Position** | **P-value** | **Position of linkage fragment** | **Adjacent known gene** | **Gene ID (MSU7.0)** | **Gene position** | **correlation coefficient r** | **How close (kb)** |
| --- | --- | --- | --- | --- | --- | --- | --- | --- | --- | --- | --- |
| **Grain length** | 0-1-2-3-4 / 0-2-1 | F35145 | 3 | 16735001 | 9.7E-18/4.9E-27 | 16704281- 18450200/ 15754601- 16737640 | ***GS3*** | None | 16729501-16735109 | 0.98 | 0 |
| **Tiller angle** | 0-1-2-3-4 | F74214 | 9 | 20805001 | 7.70E-11 | 18577801- 21363080 | ***TAC1*** | LOC_Os09g35980 | 20734724 - 20731589 | 1.00 | -70 |
| **Flag leaf width** | 0-1-2-3-4 / 0-1-0 | F45261 | 4 | 30900001 | 3.04E-13 | 30254881- 31483680/ 30670881- 31479840 | ***NAL1****; qFLW4; LSCHL4; NAL5* | LOC_Os04g52479 | 31203525 - 31214741 | 0.93 | 296 |
| **Days to flowering** | 0-1-2-3-4 | F64241 | 7 | 29085001 | 5.28546E-11 | 28869961- 29714760 | ***DTH7****; Ghd7.1; OsPRR37* | LOC_Os07g49460 | 29616705 - 29629223 | 0.91 | 515 |

Note: In the “How close (kb)” column, the positive numbers mean the downstream distance between the known gene and identified locus from GWAS, the negative numbers mean upstream distance between the known gene and identified locus from GWAS; The correlation coefficient r values were generated by using Pearson correlation test.

**Table S9.** Summary of tested gene and pairwise gene combination numbers used in epistasis analysis and the identified epistatic gene pair numbers with their corresponding contributions to a given trait.

| **Traits** | **Tested loci number** | **Pairwise gene combination number** | **A by A** | **A by D** | **D by A** | **D by D** | **All** |
| --- | --- | --- | --- | --- | --- | --- | --- |
| **Days to flowering** | 13 | 78 | 15 (19.23%) | 5 (6.41%) | 9 (11.54%) | 6 (7.69%) | 35 (44.87%) |
| **Plant height** | 16 | 120 | 8 (6.67%) | 5 (4.17%) | 7 (5.83%) | 4 (3.33%) | 24 (20.00%) |
| **Flag leaf length** | 8 | 28 | 4 (14.29%) | 4 (14.29%) | 4 (14.29%) | 3 (10.71%) | 15 (53.57%) |
| **Flag leaf width** | 16 | 120 | 17 (14.17%) | 6 (5.00%) | 7 (5.83%) | 2 (1.67%) | 32 (26.67%) |
| **Flag leaf angle** | 15 | 105 | 4 (3.81%) | 13 (12.38%) | 11 (10.48%) | 11 (10.48%) | 39 (37.14) |
| **Tiller angle** | 21 | 210 | 38 (18.10%) | 22 (10.48%) | 29 (13.81%) | 13 (6.19%) | 102 (48.57%) |
| **Stem diameter** | 12 | 66 | 4 (6.06%) | 3 (4.55%) | 9 (13.64%) | 1 (1.52%) | 17 (25.76%) |
| **Tiller number** | 19 | 171 | 2 (1.17%) | 6 (3.51%) | 18 (10.53%) | 10 (5.85%) | 36 (21.05%) |
| **Panicle length** | 15 | 91 | 9 (9.89%) | 3 (3.30%) | 7 (7.69%) | 1 (1.10%) | 20 (21.98%) |
| **Primary branch number per panicle** | 20 | 190 | 22 (11.58%) | 13 (6.84%) | 20 (10.53%) | 3 (1.58%) | 58 (30.53%) |
| **Secondary branch number per panicle** | 13 | 78 | 1 (1.28%) | 4 (5.13%) | 5 (6.41%) | 7 (8.97%) | 17 (21.79%) |
| **Spikelet number per panicle** | 12 | 66 | 2 (3.03%) | 3 (4.55%) | 4 (6.06%) | 0 | 9 (13.64%) |
| **Grain number per panicle** | 16 | 120 | 7 (5.83%) | 9 (7.50%) | 7 (5.83%) | 6 (5.00%) | 29 (24.17%) |
| **Spikelet density per panicle** | 5 | 10 | 1 (10%) | 1 (10%) | 1 (10%) | 0 | 3 (30%) |
| **Fertility** | 13 | 78 | 7 (8.97%) | 5 (6.41%) | 2 (2.56%) | 5 (6.41%) | 19 (24.36%) |
| **Thousand kernel weight** | 16 | 120 | 27 (22.50%) | 9 (7.50%) | 15 (12.50%) | 4 (3.33%) | 55 (45.83%) |
| **Yield** | 10 | 45 | 0 | 1 (2.22%) | 1 (2.22%) | 0 | 2 (4.44%) |
| **Grain length** | 29 | 406 | 48 (11.82%) | 40 (9.85%) | 46 (11.33%) | 34 (8.37%) | 168 (41.38%) |
| **Grain width** | 22 | 231 | 22 (9.52%) | 15 (6.49%) | 24 (10.39%) | 24 (10.39%) | 85 (36.80%) |
| **Grain length to width ratio** | 16 | 120 | 20 (16.67%) | 10 (8.33%) | 13 (10.83%) | 5 (4.17%) | 48 (40.00%) |
| **Biomass** | 9 | 36 | 1 (2.78%) | 1 (2.78%) | 0 | 1 (2.78%) | 3 (8.33%) |
| **Total** | 316 | 2489 | 259 (10.41%) | 178 (7.15%) | 239 (9.60%) | 140 (5.62%) | 816 (32.78%) |

Supporting Material. GWAS plots of all 21 investigated traits.


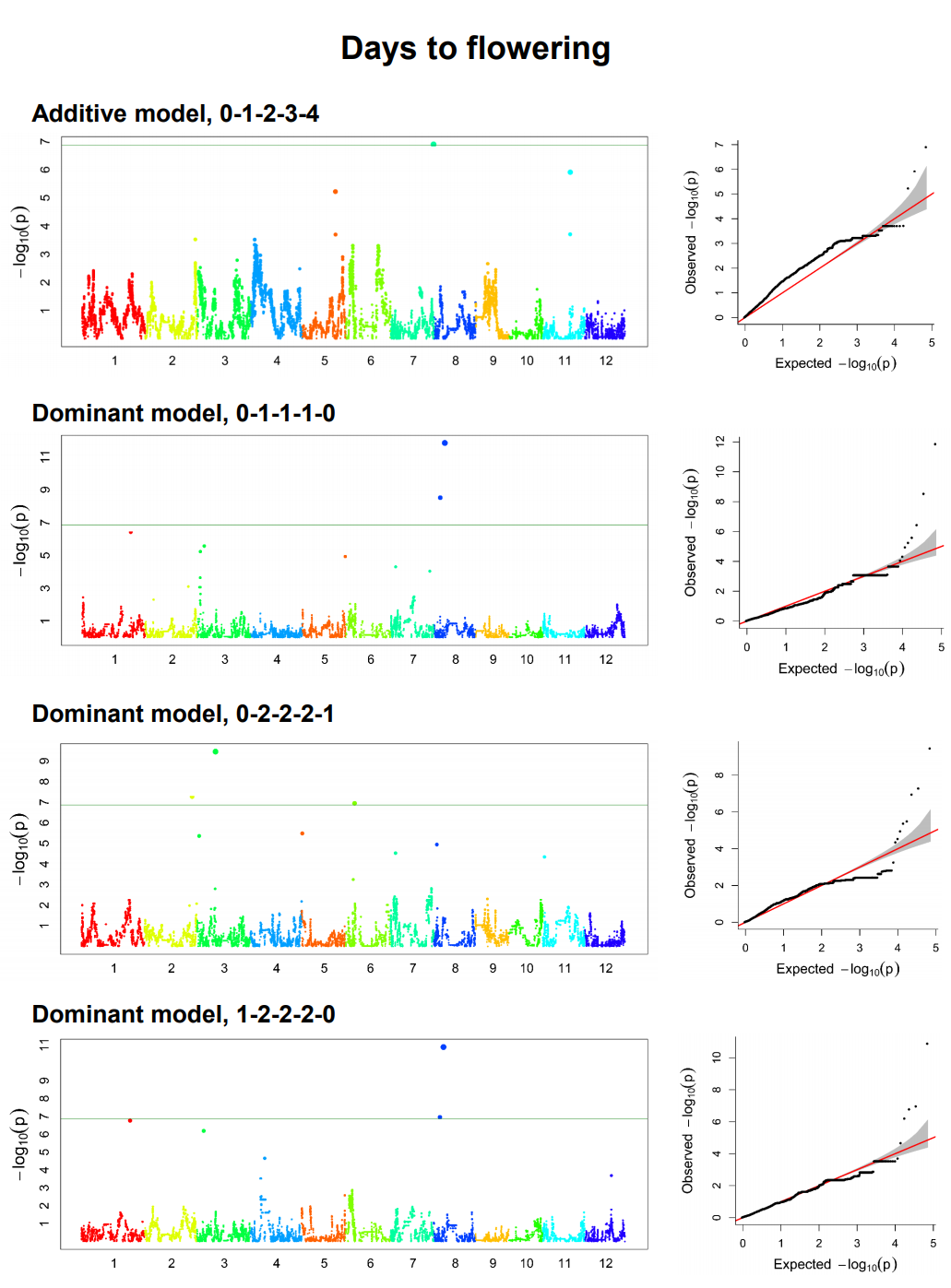


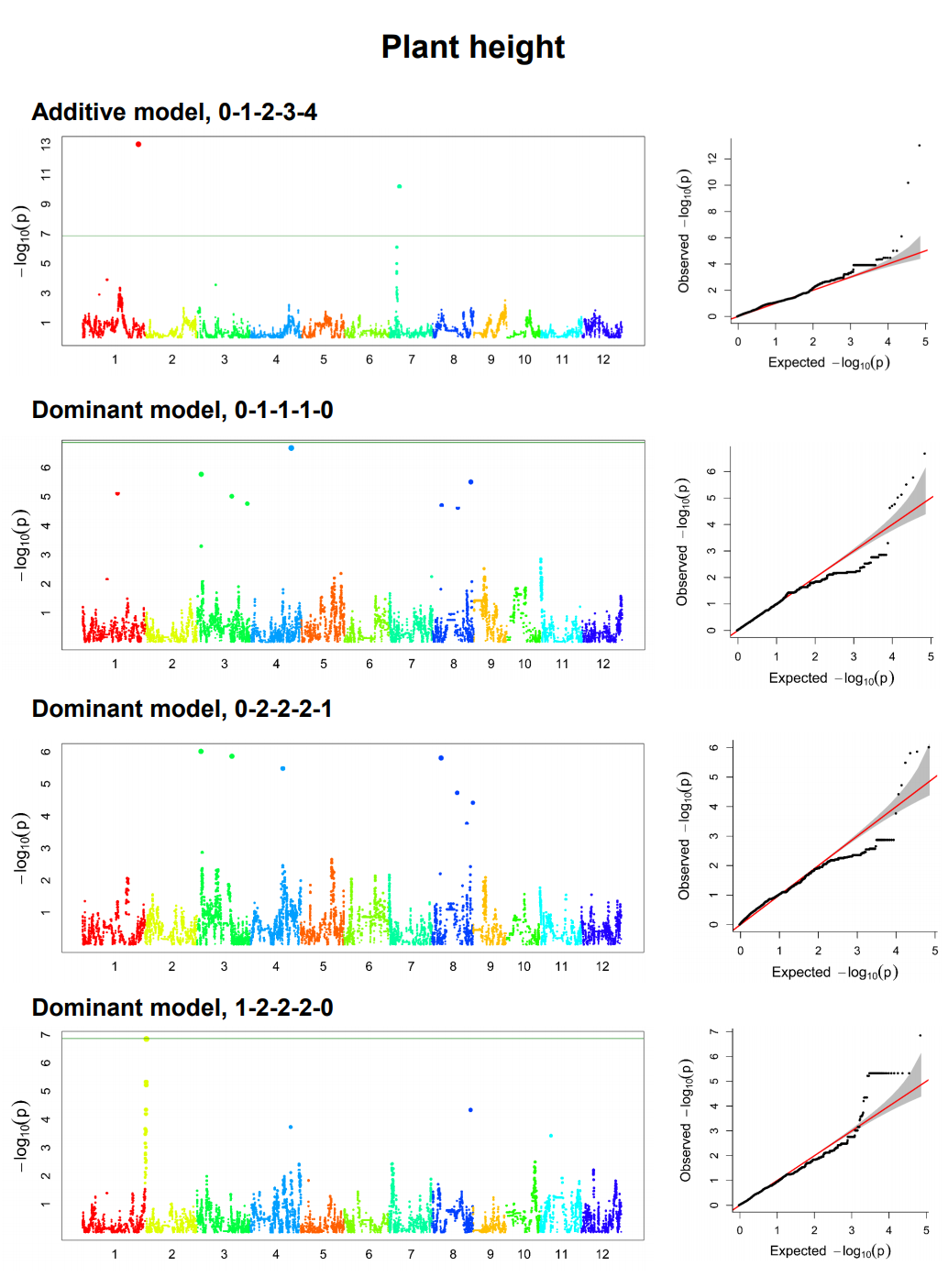


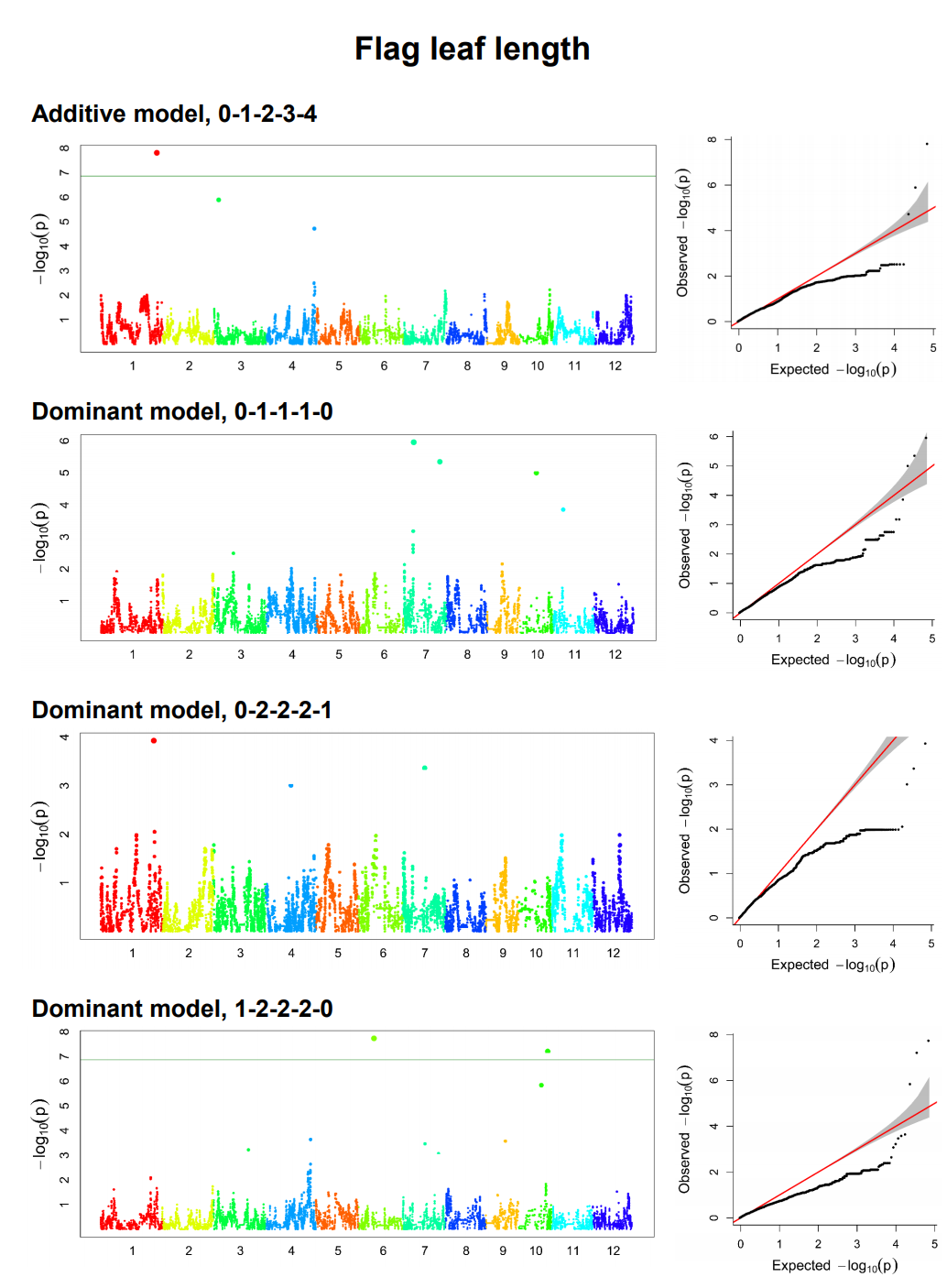


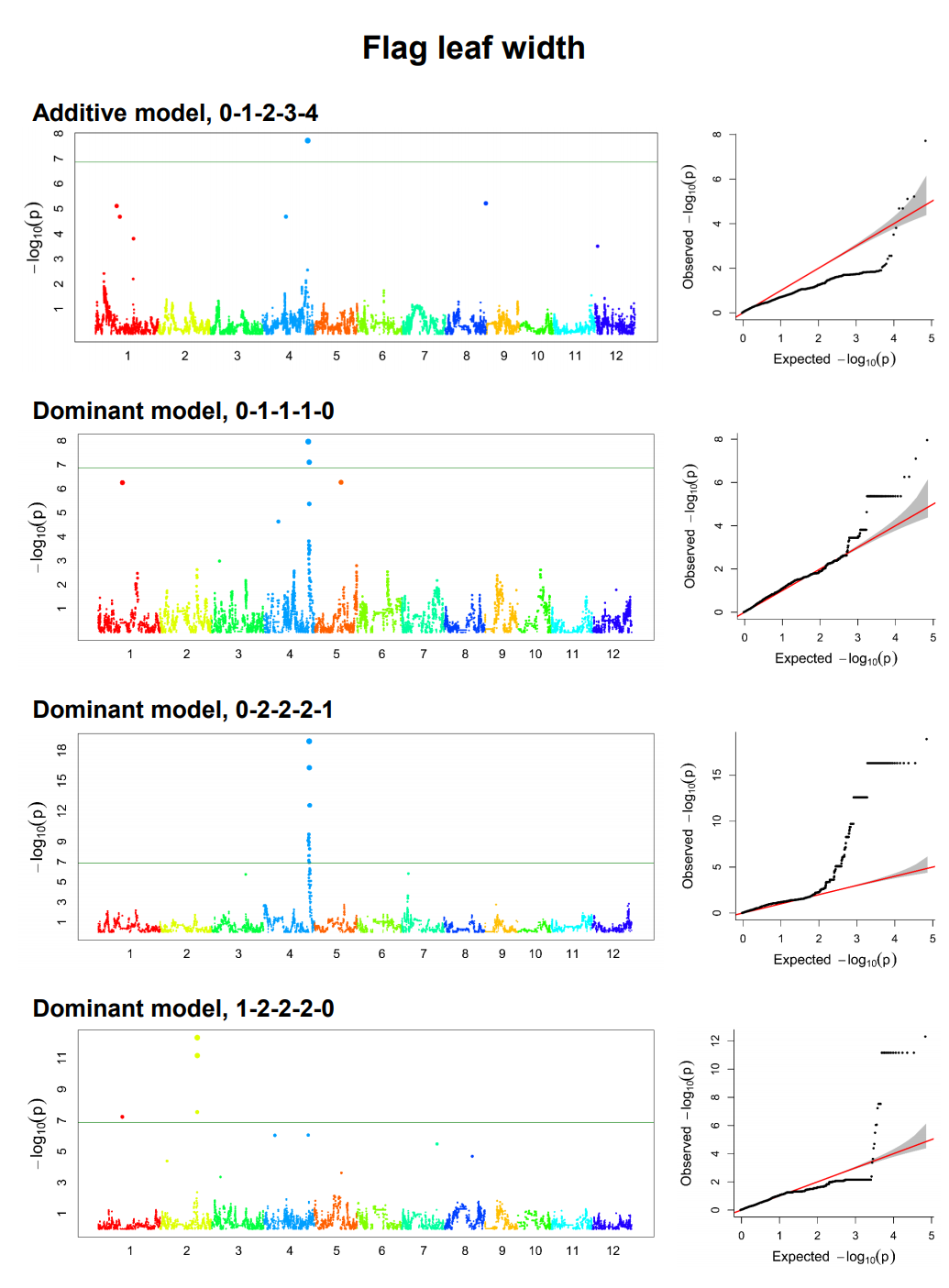


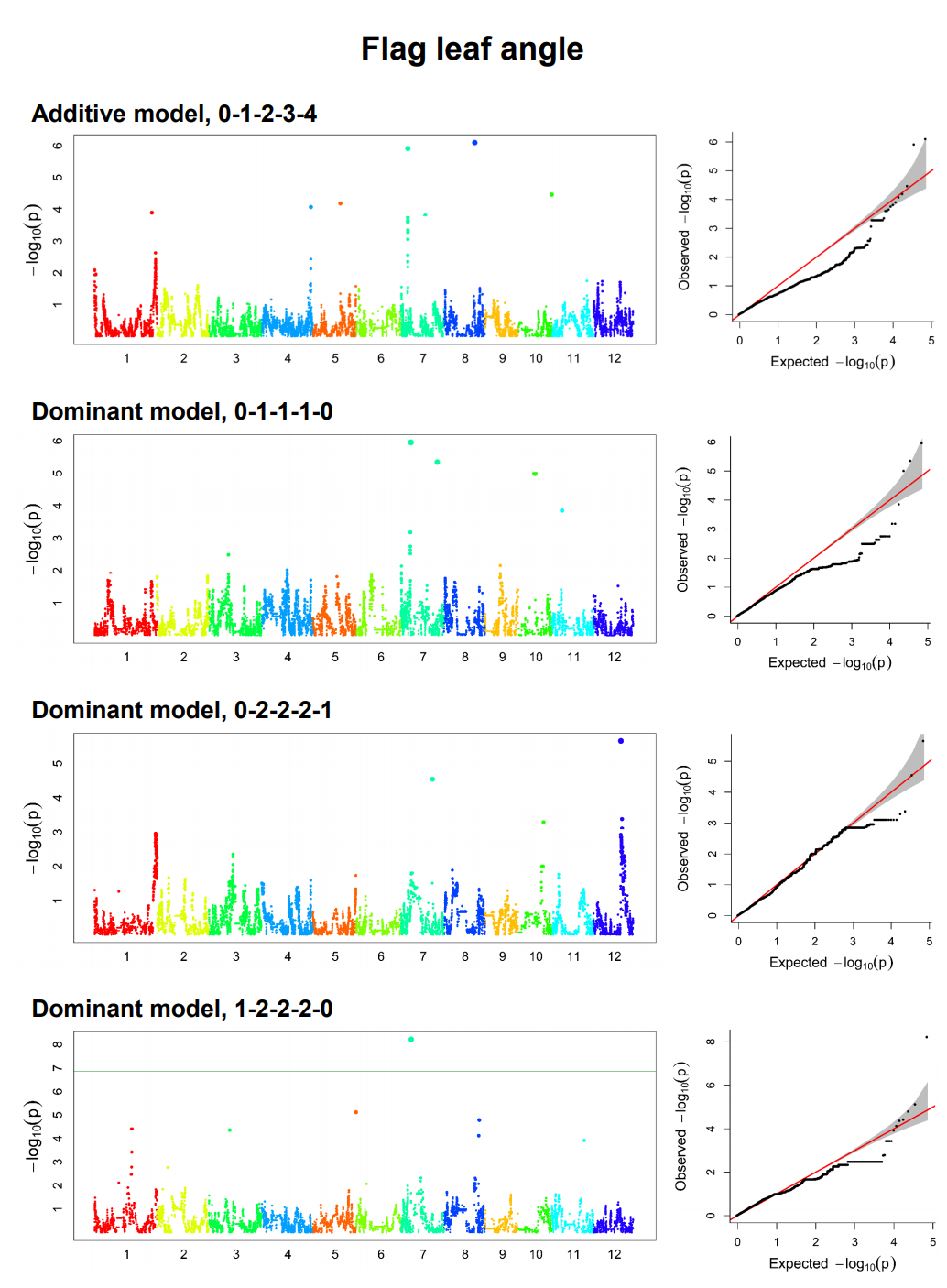


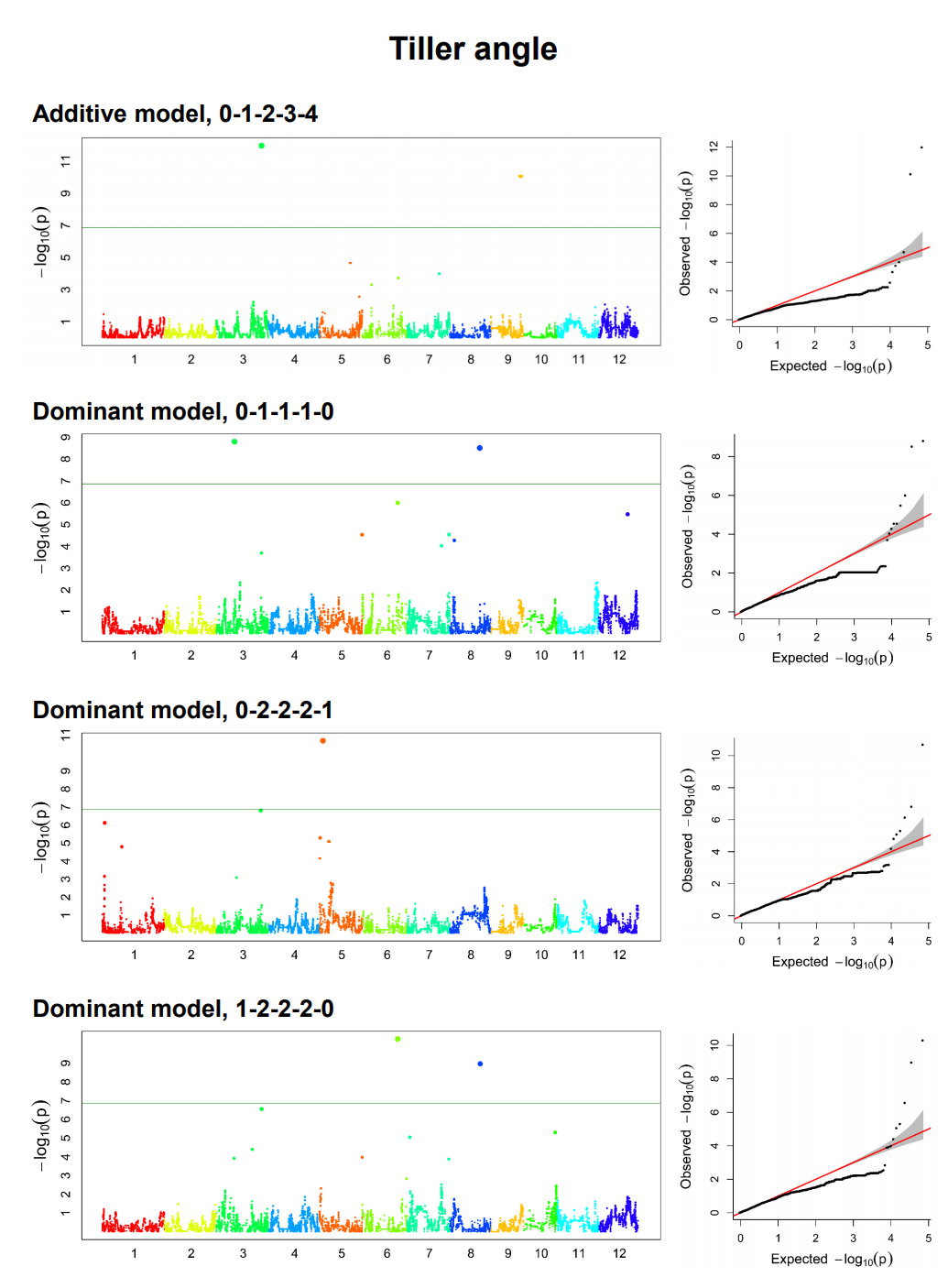


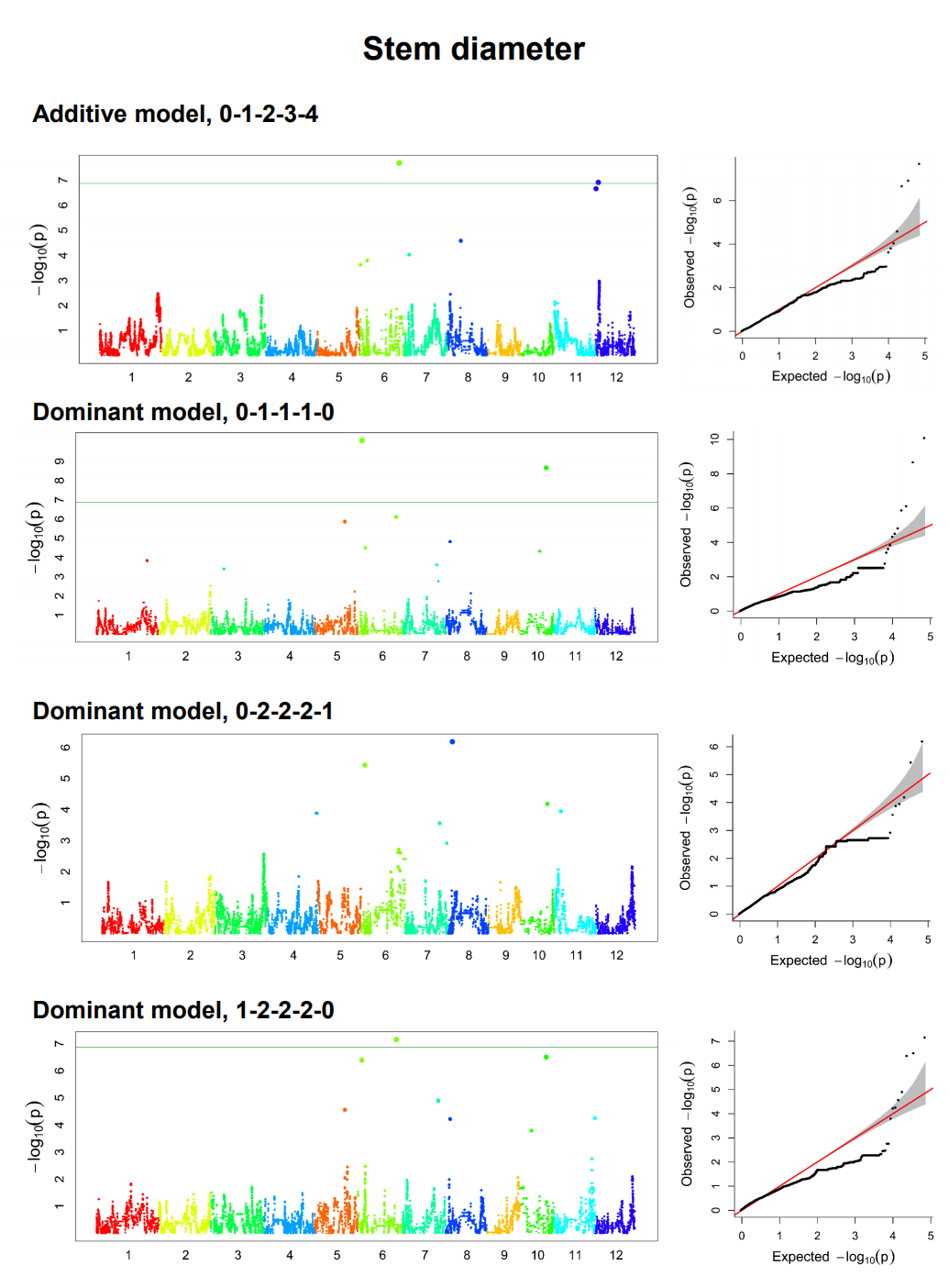


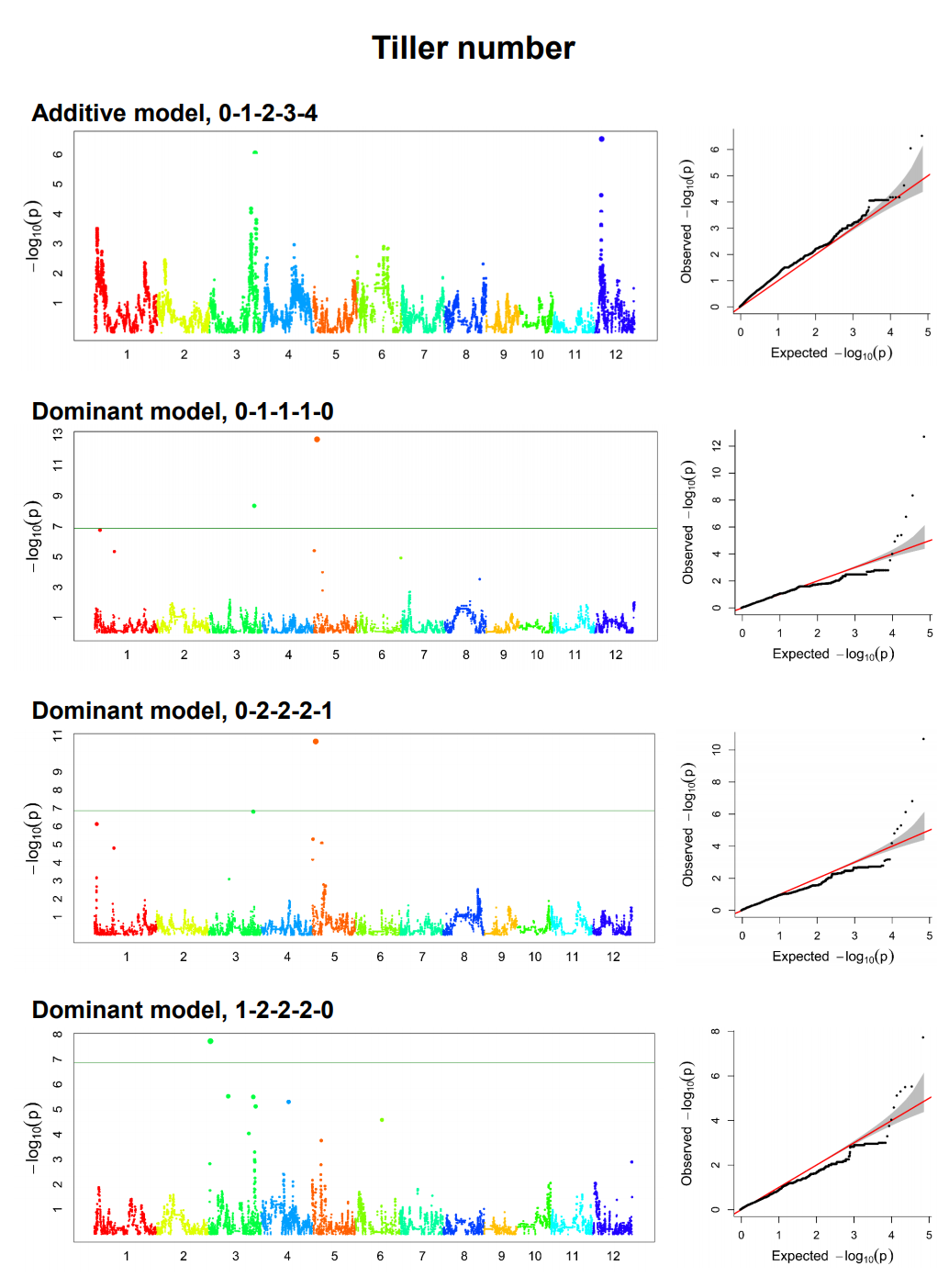


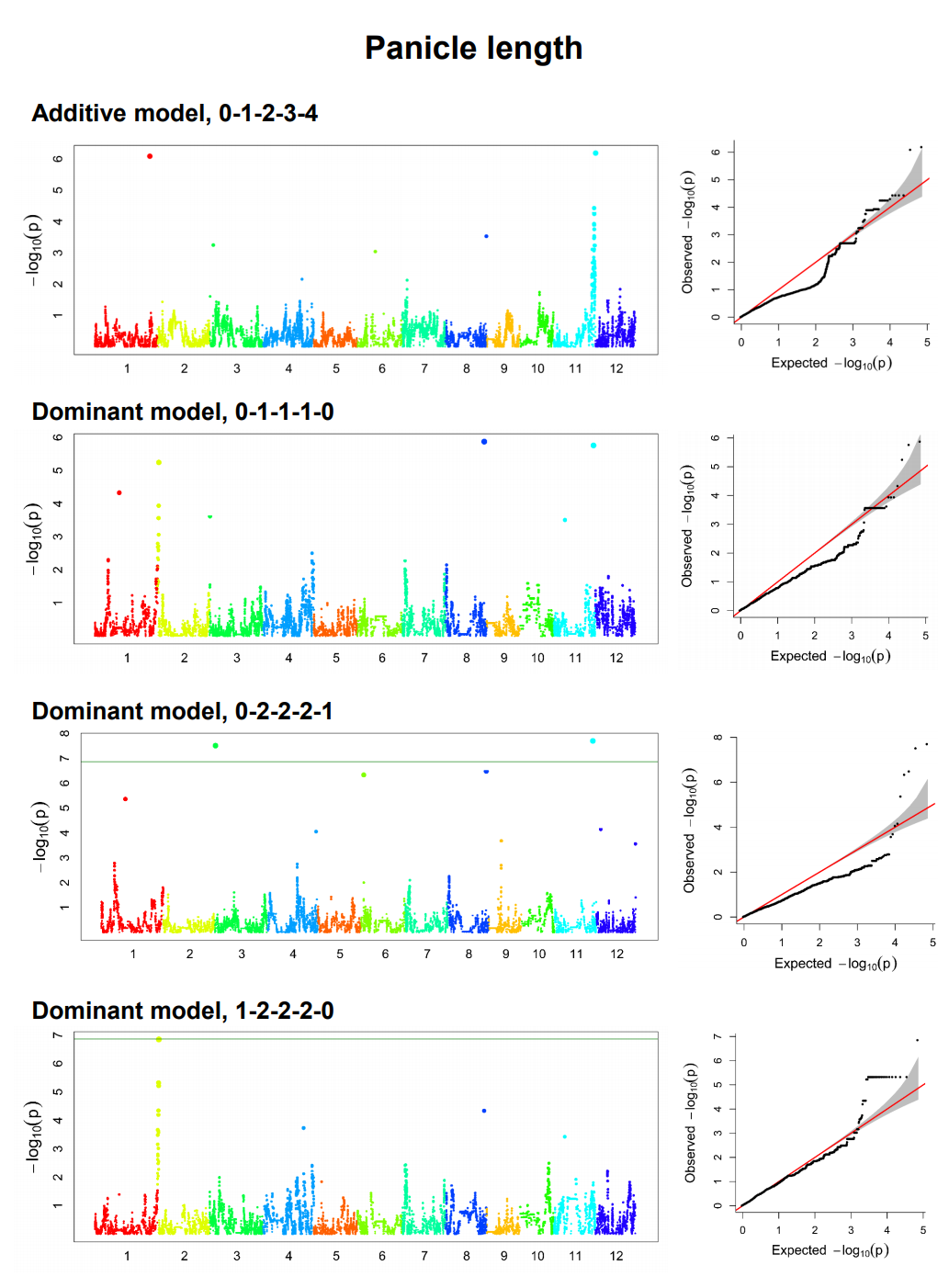


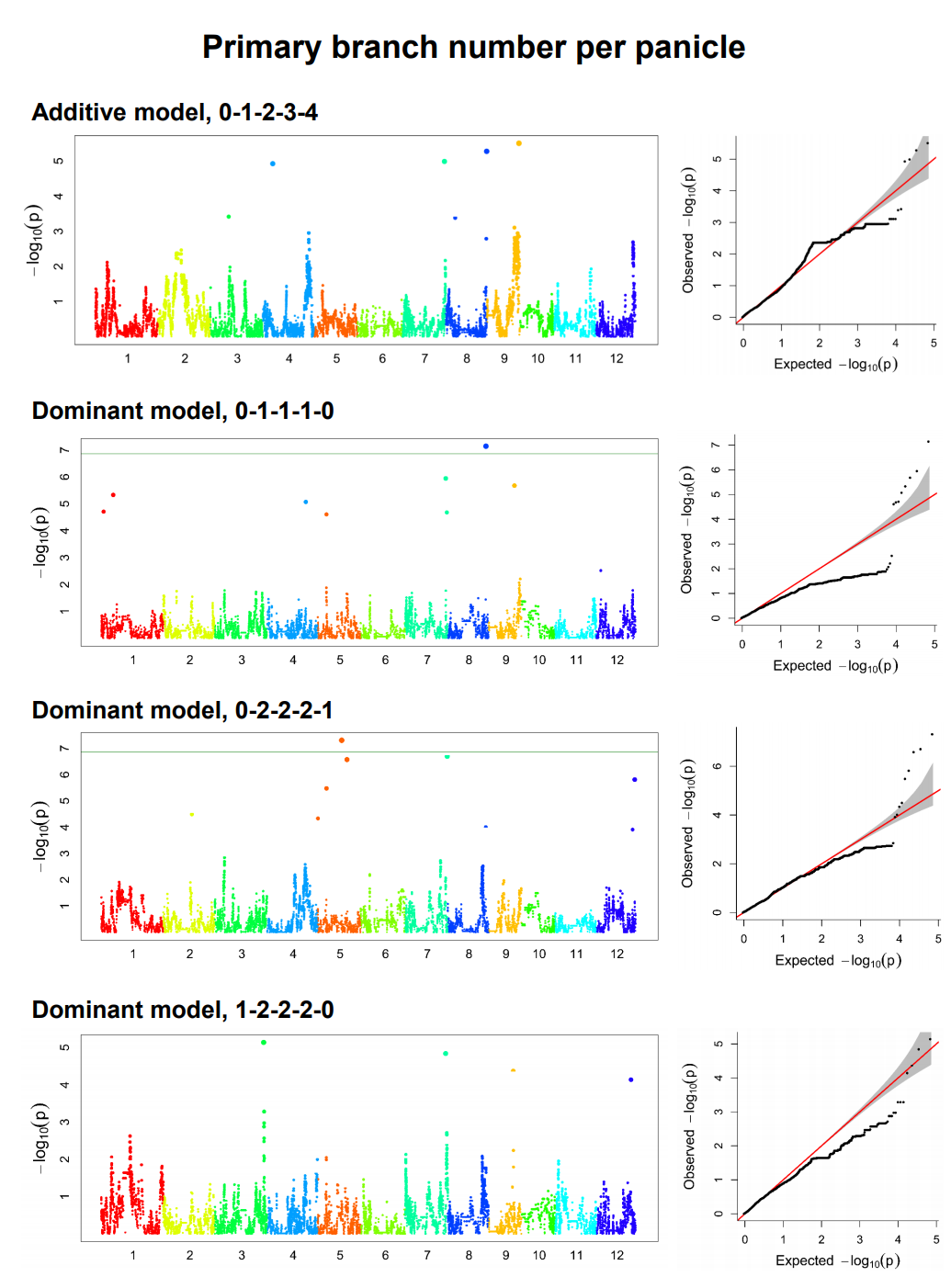


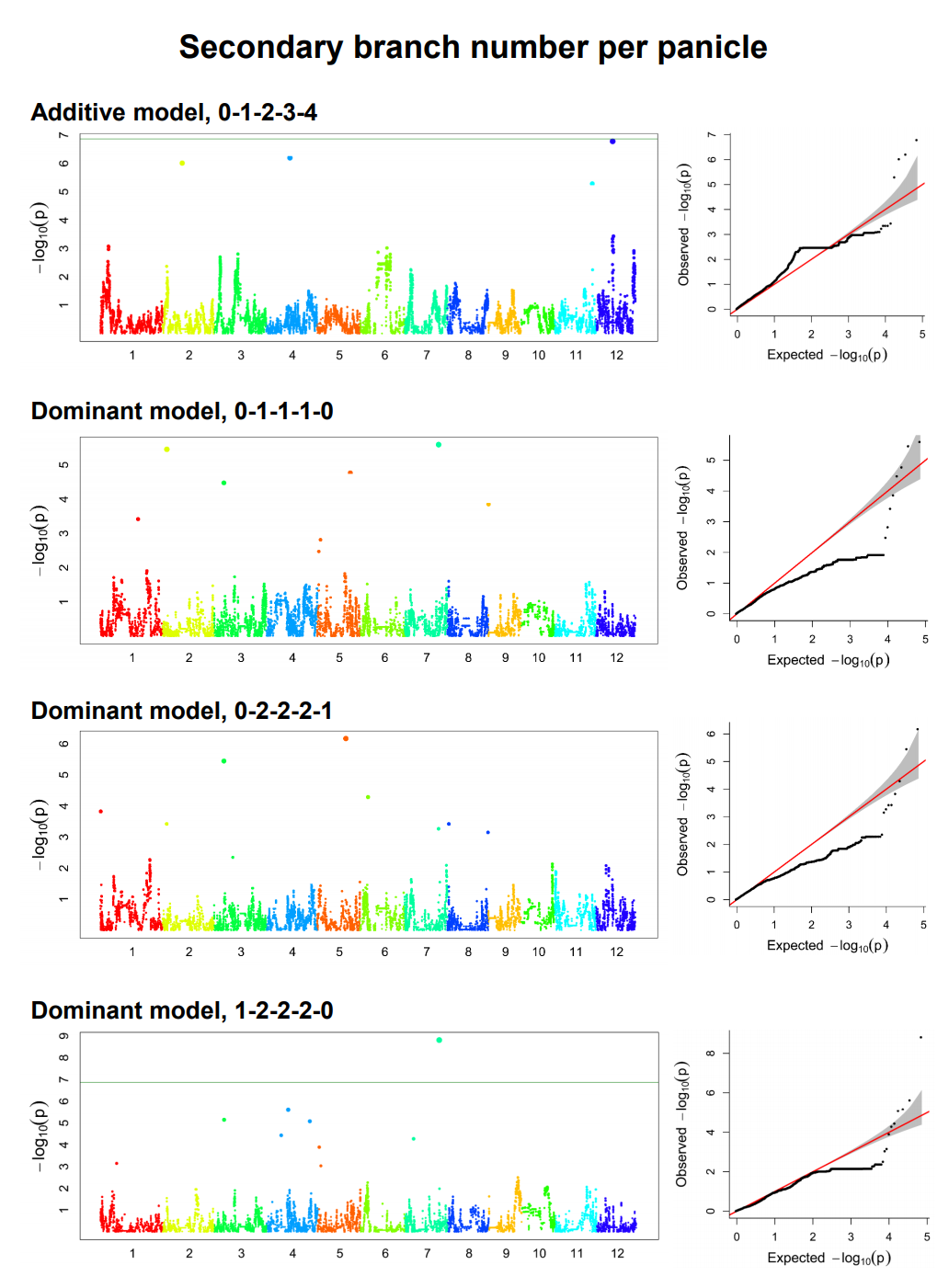


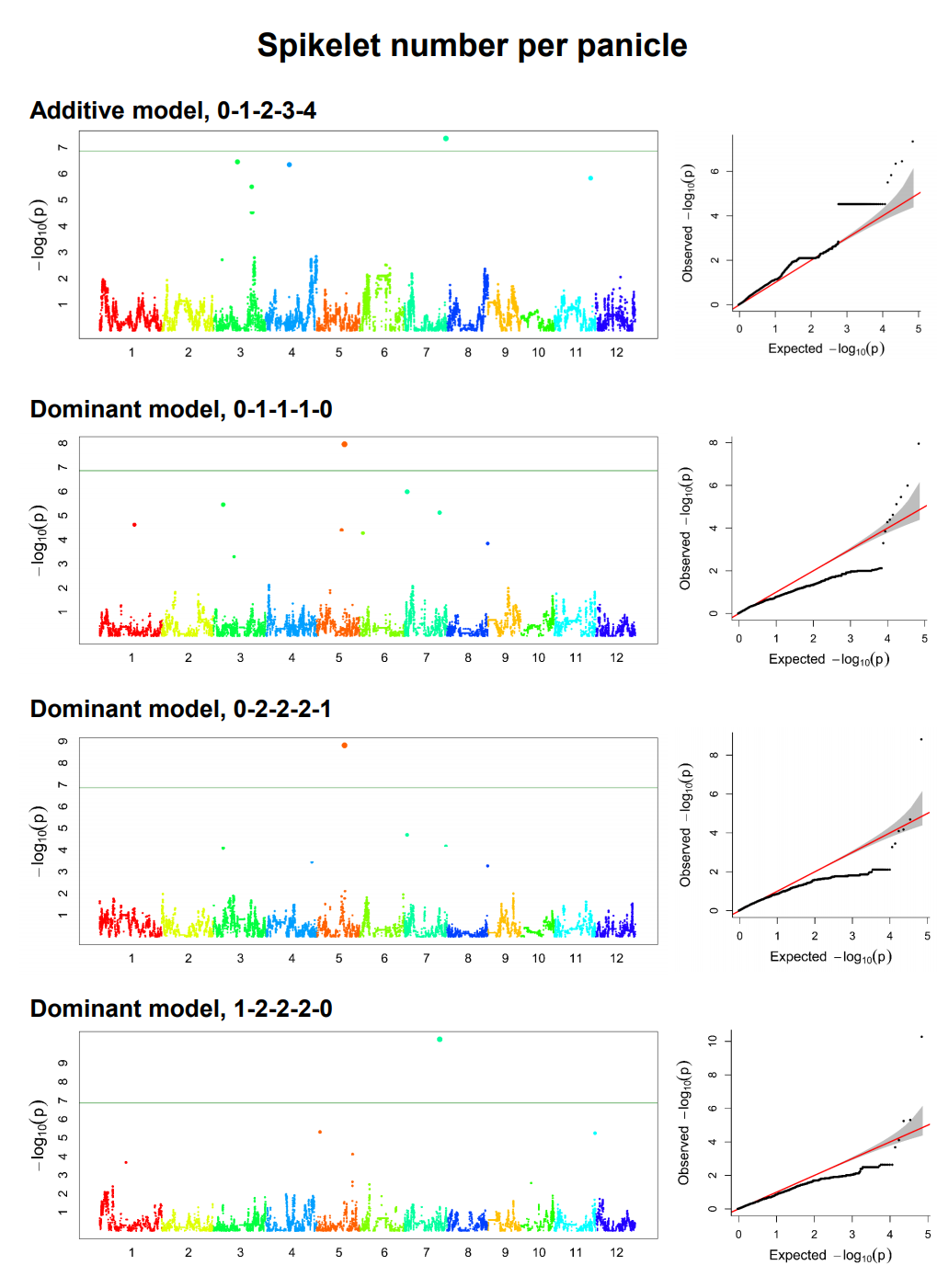


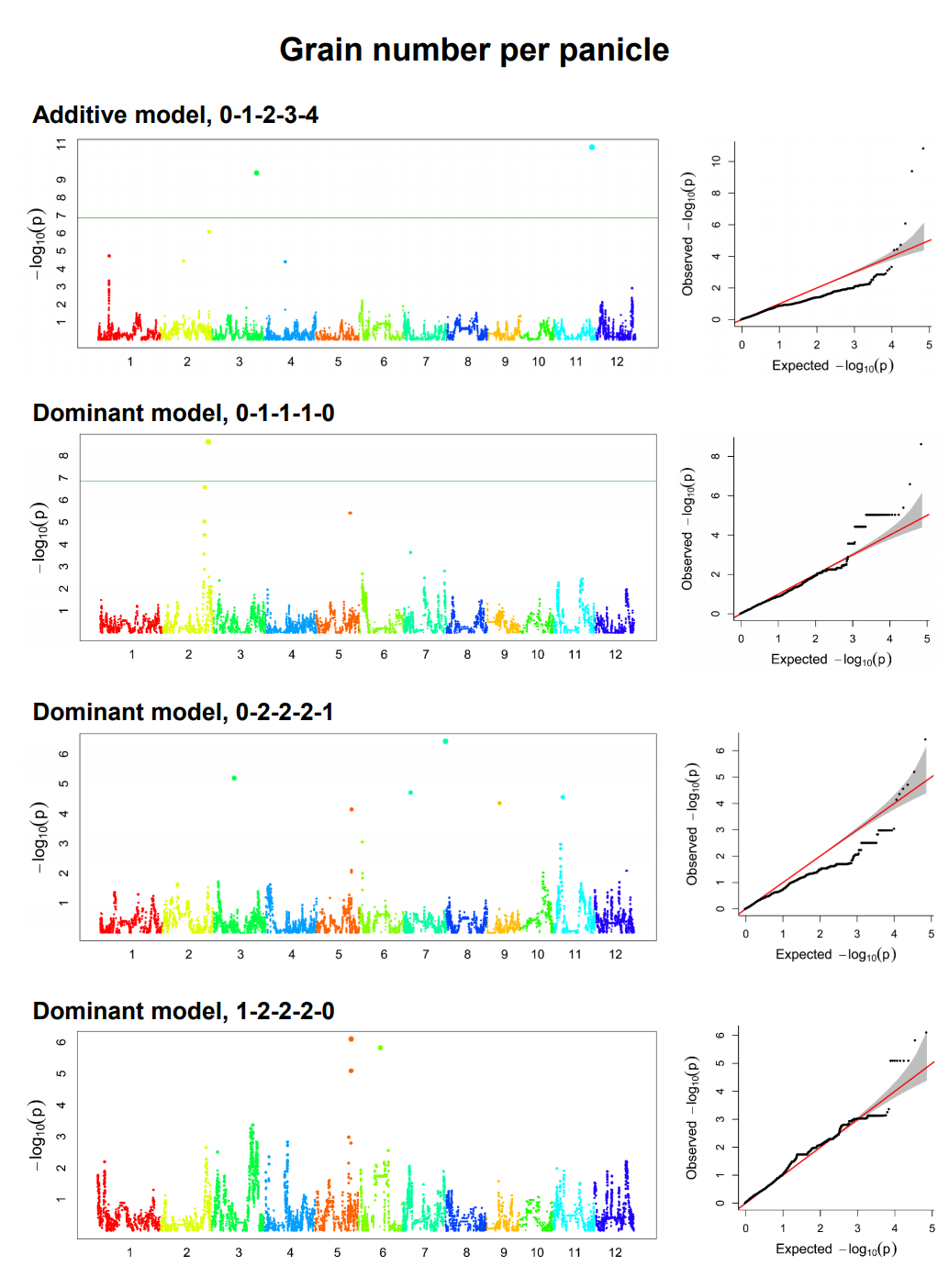


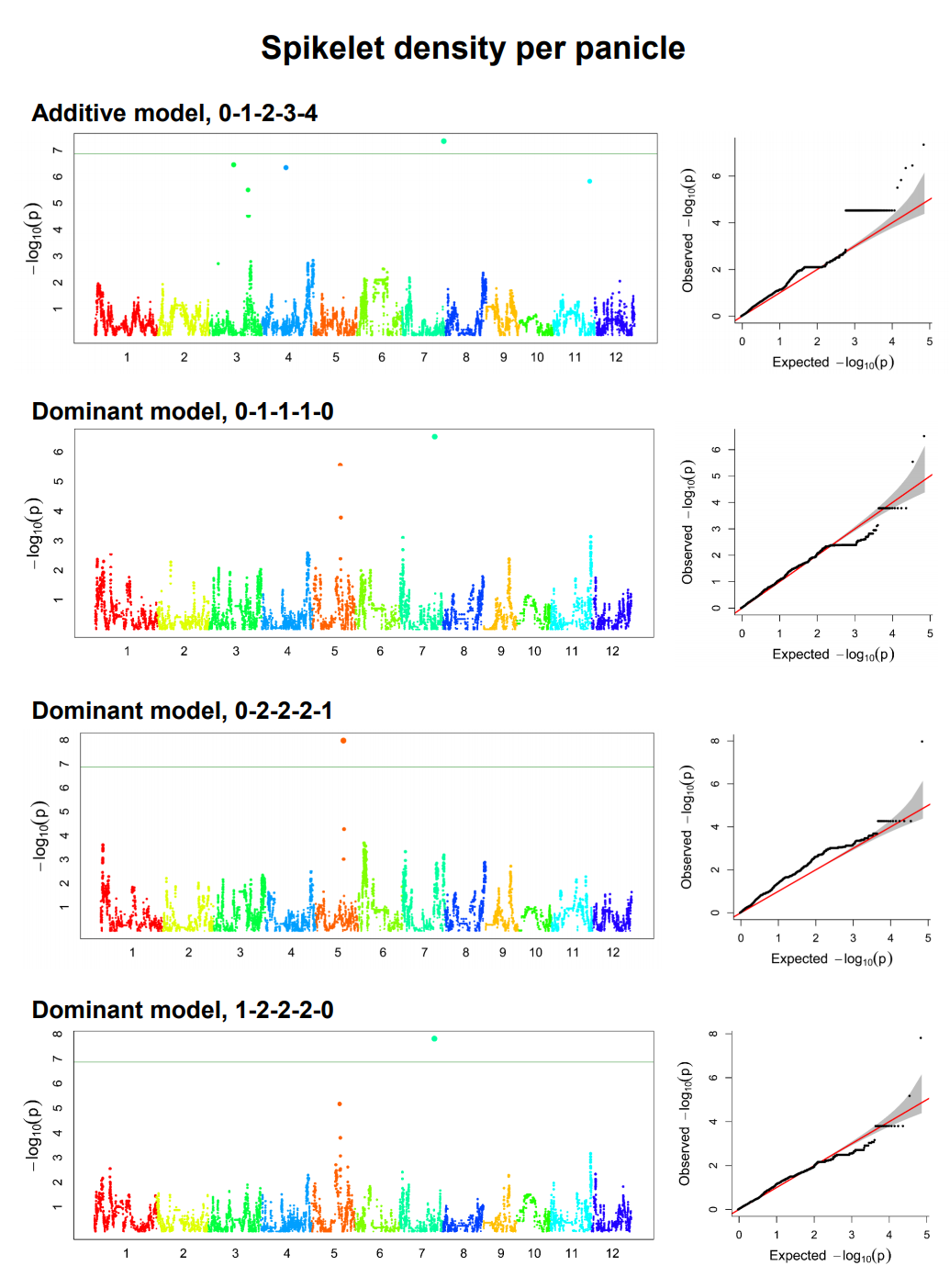


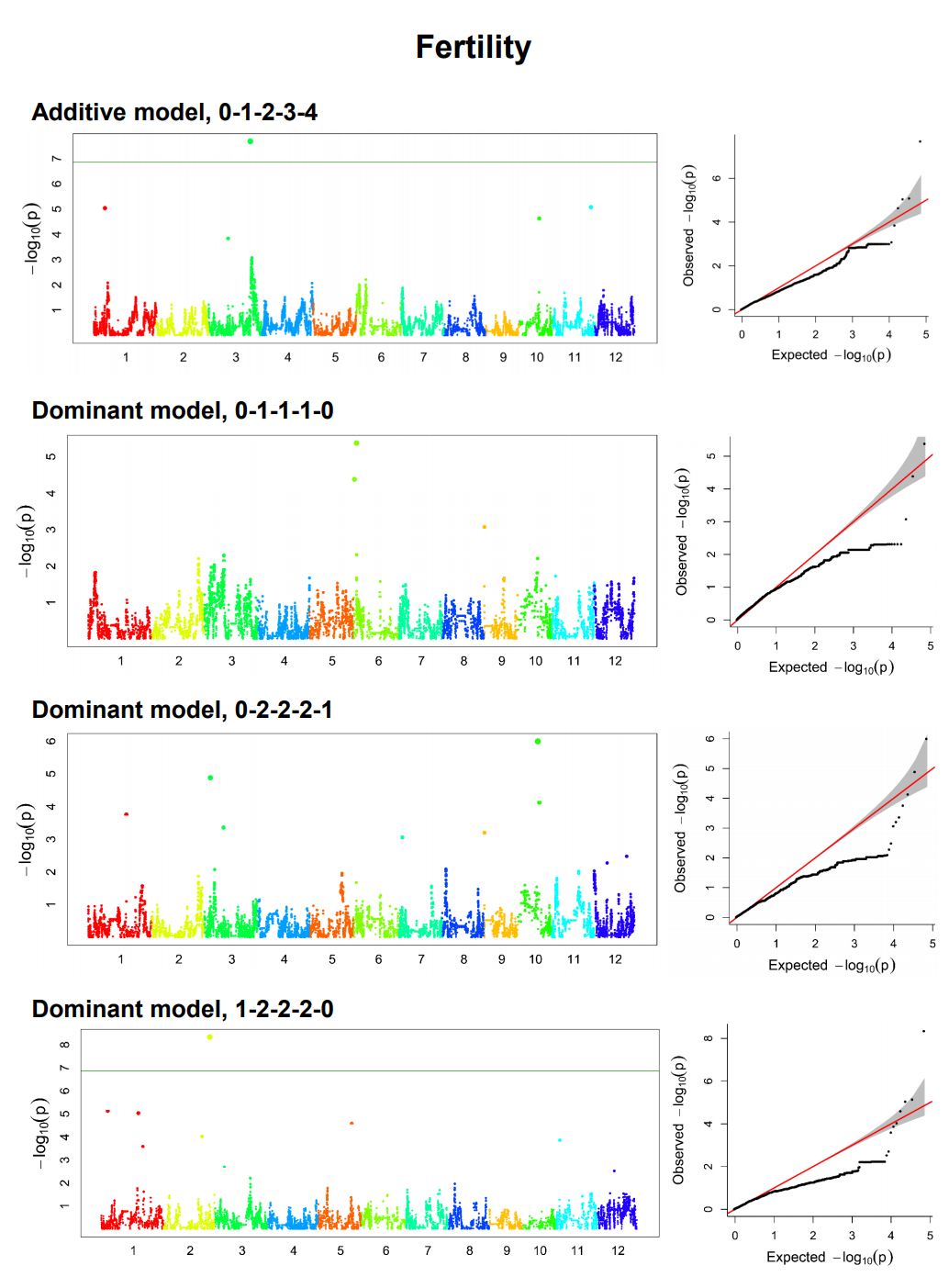


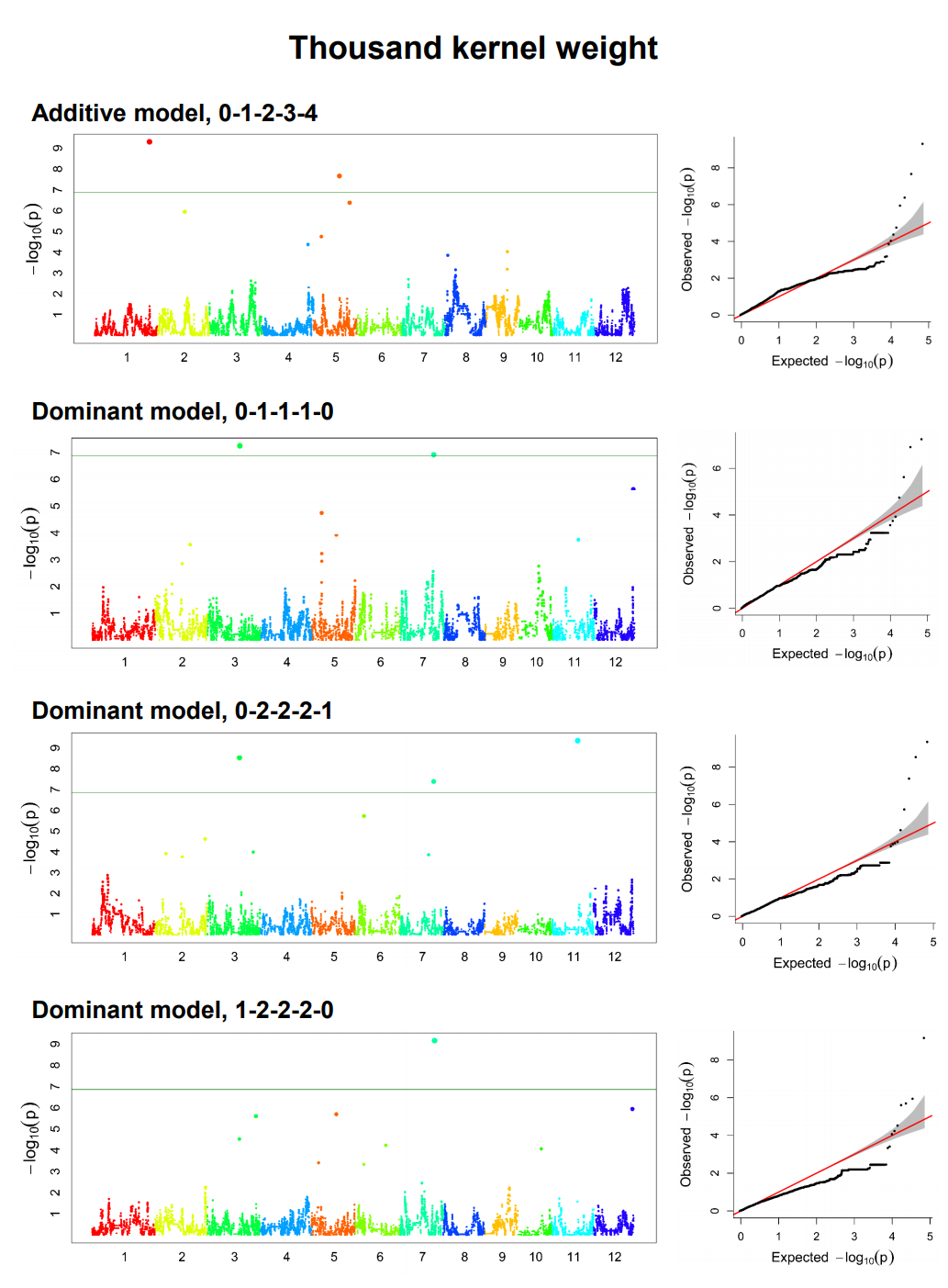


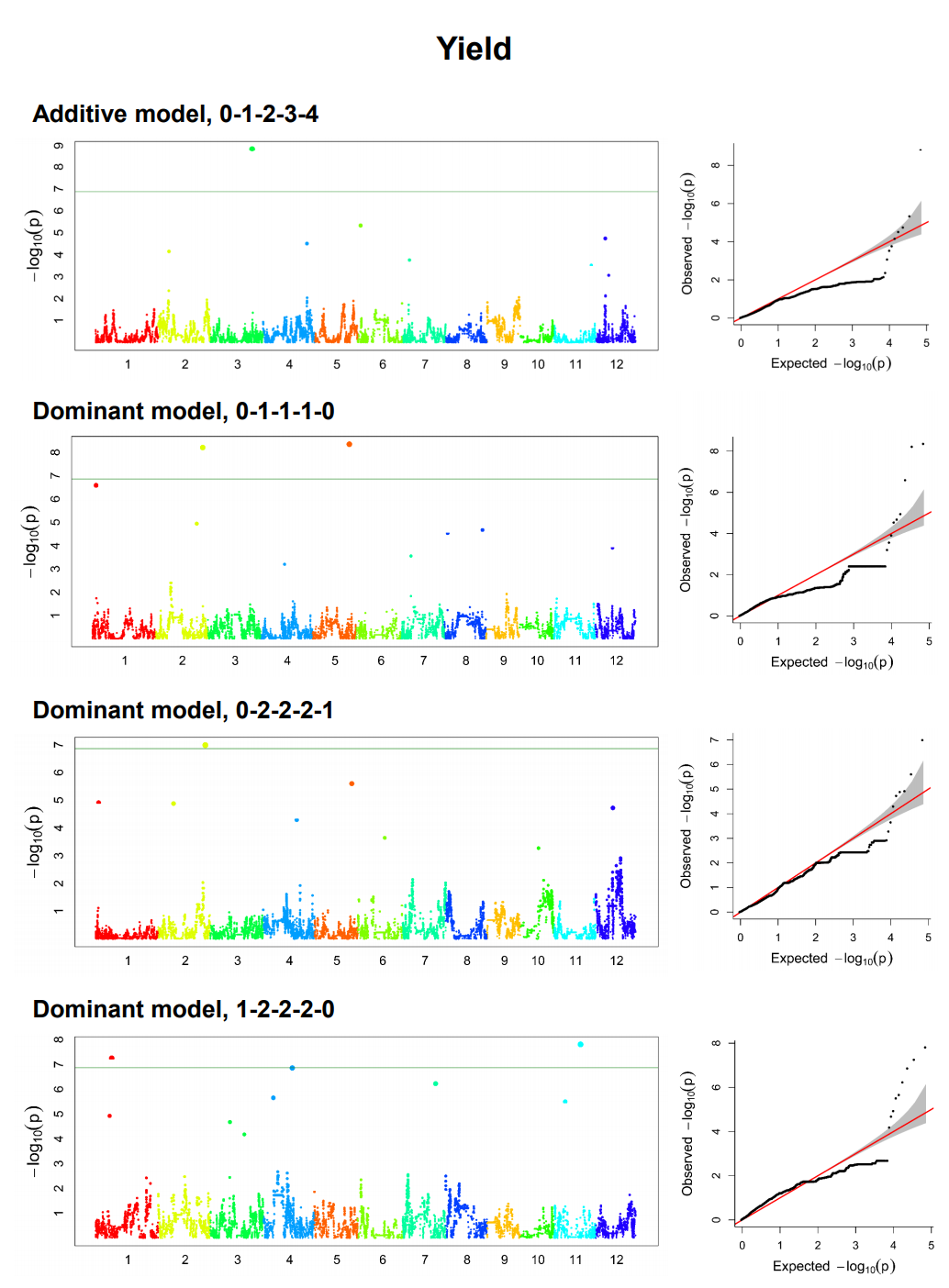


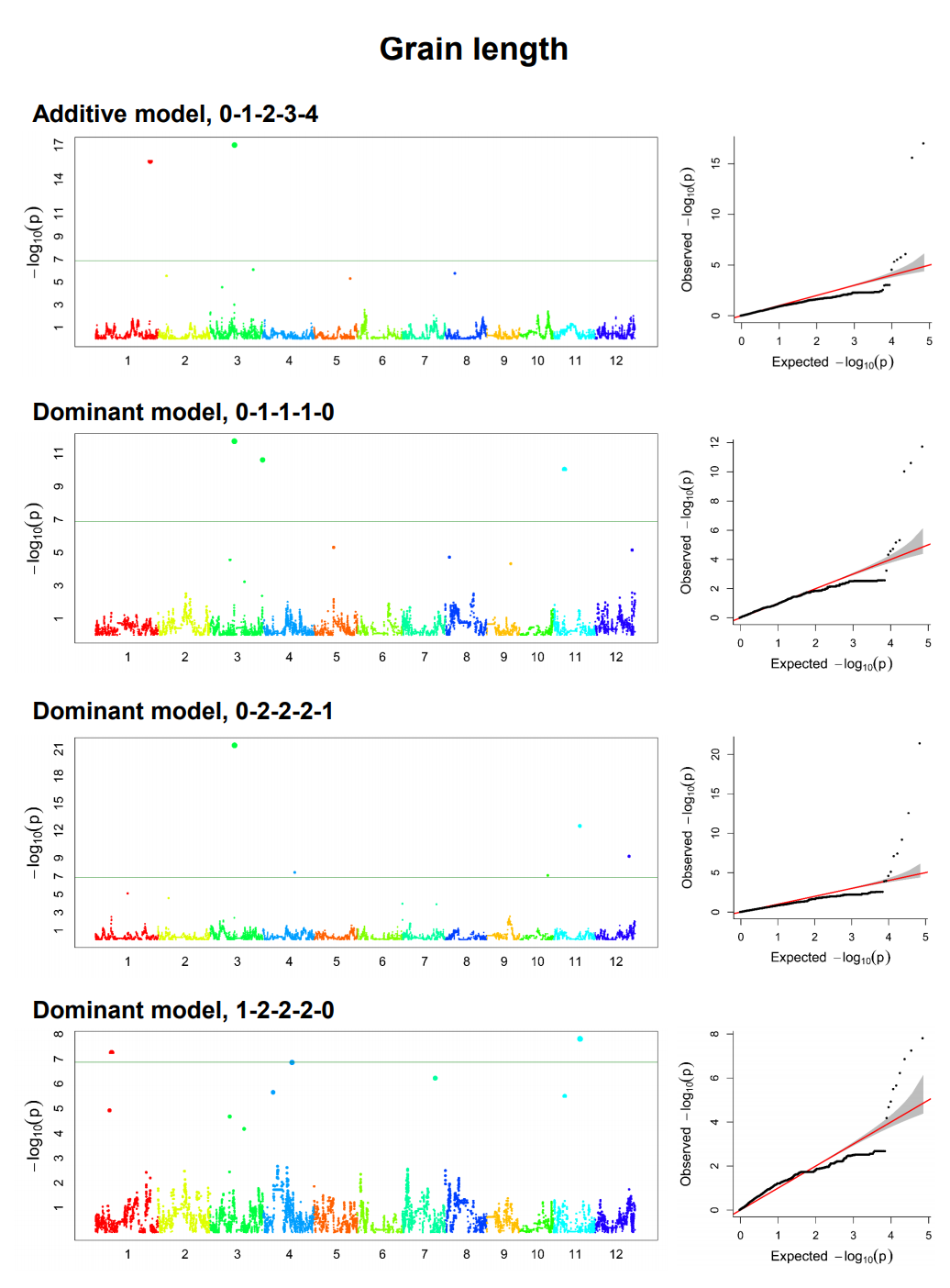


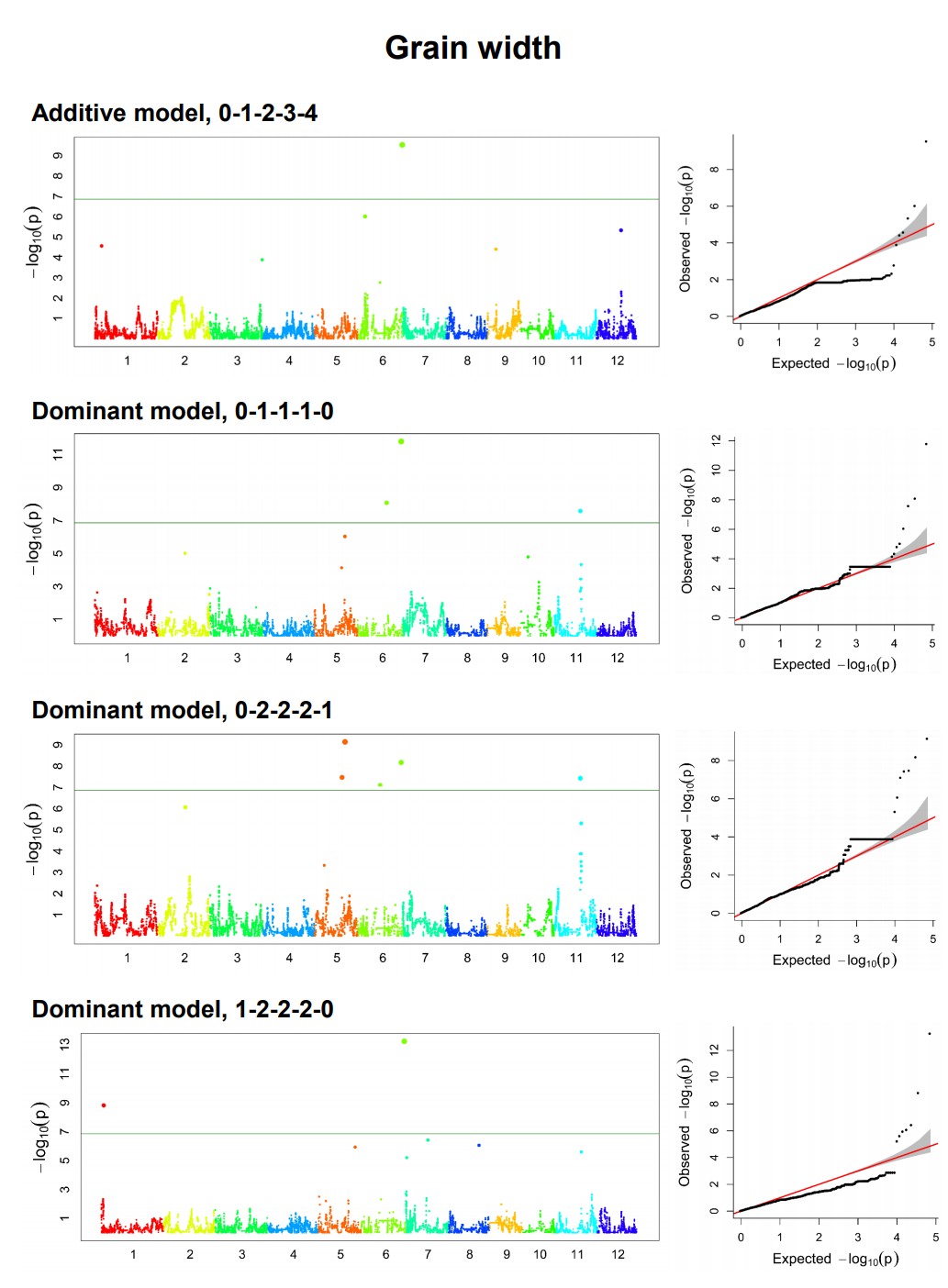


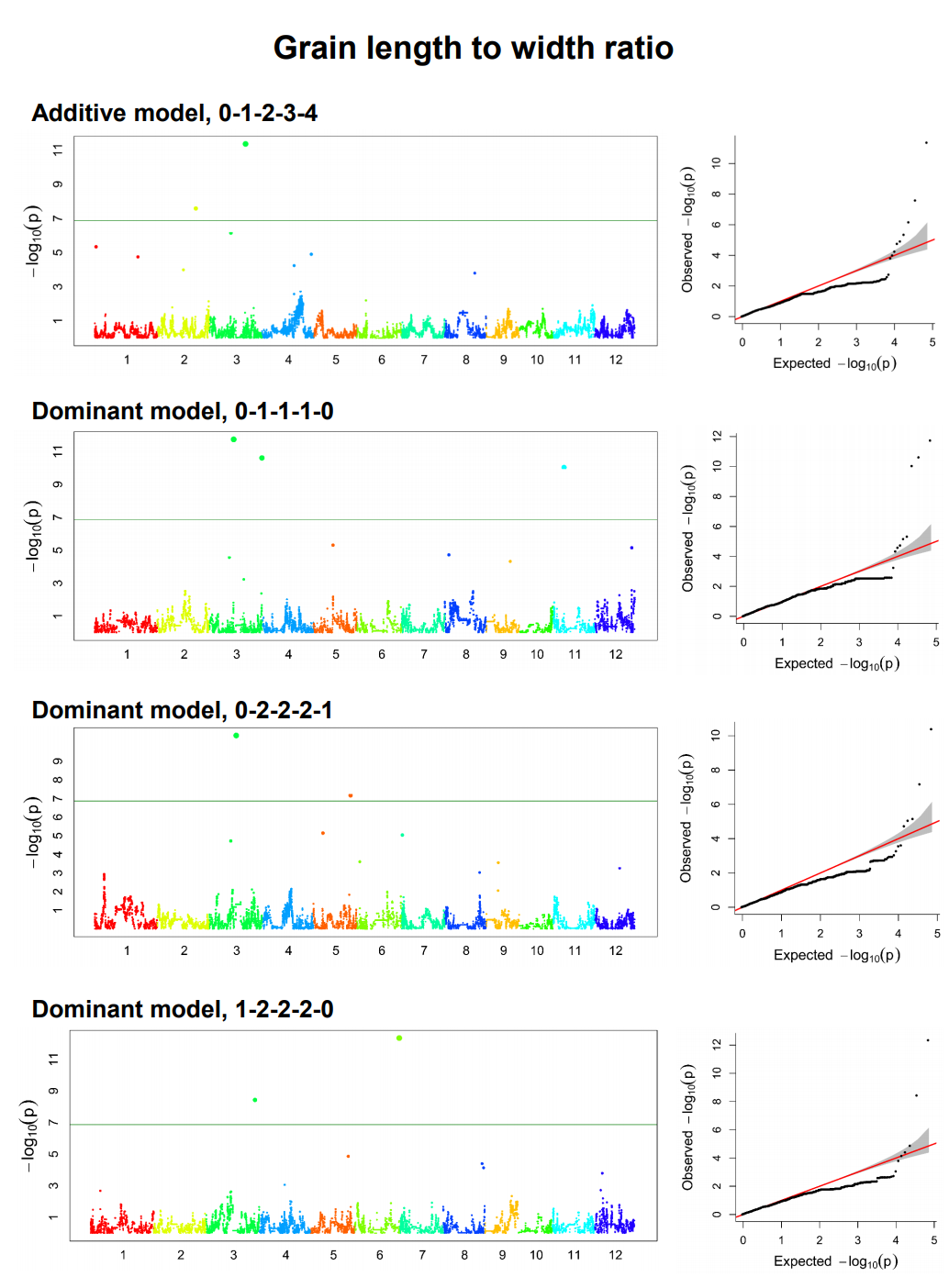


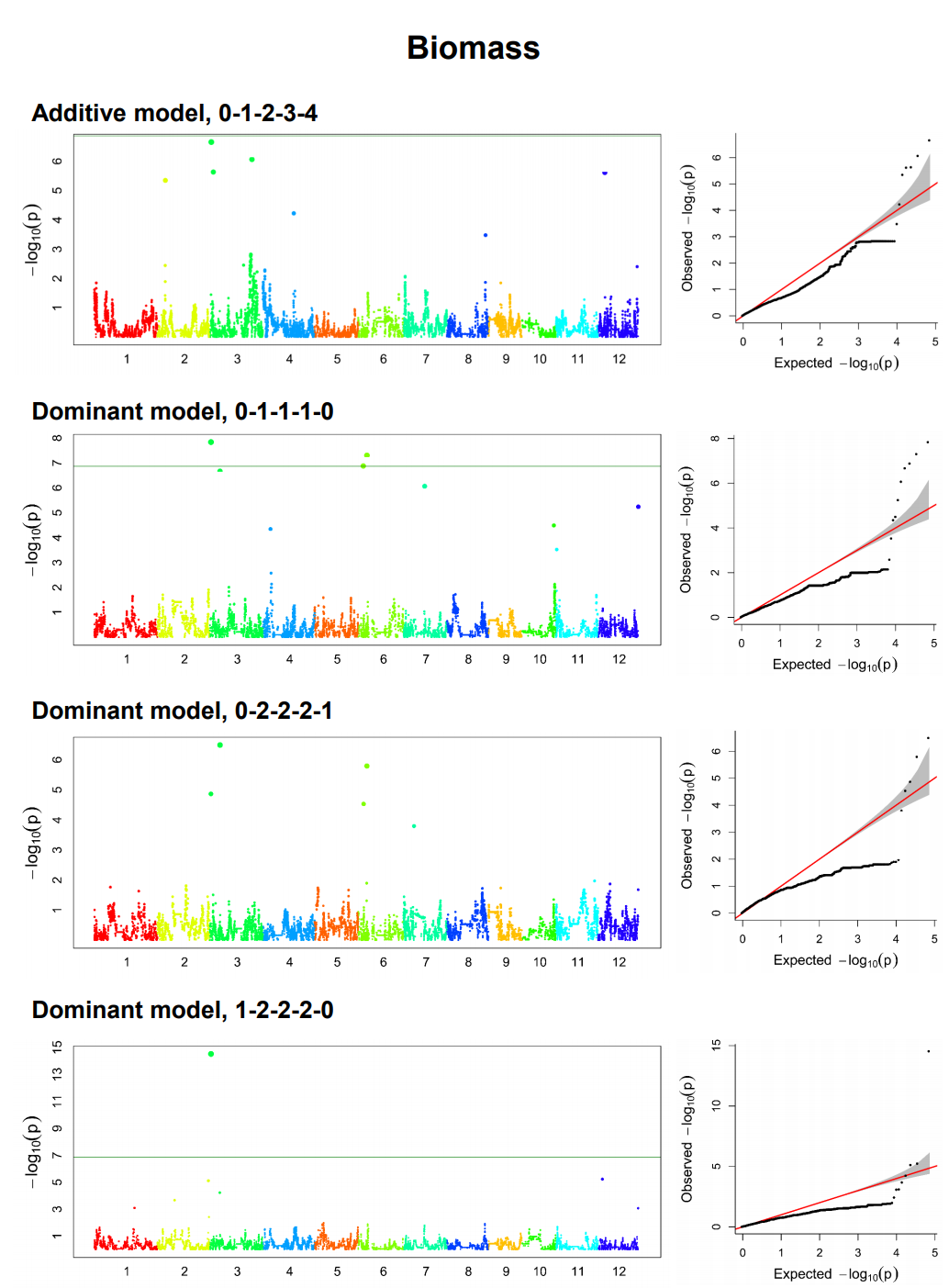


Dataset S1 (separate file). Summary of phenotypic data for each investigated population.

Dataset S2 (separate file). Pedigree and phenotypic data of the 202 tetraploid individuals used in this study.

Dataset S3 (separate file). Summary of the 31 chromosomal segments with related information, including physical location and the number of cytonuclear molecular interactional genes within them. Dataset S3 includes Dataset S3 A-D.

Dataset S4 (separate file). Summary of the GWAS results from both additive and dominant models and the number of non-redundant adjacent known genes for each associated signal. Dataset S4 includes Dataset S4 A-V.

Dataset S5 (separate file). Epistatic effect detection on identified loci from GWAS. Dataset S5 includes Dataset S5 A-W.

Dataset S6 (separate file). Coding used in the F∞ method for identifying locus pairs showing di-genic epistasis.
